# Neurochemical and Behavioral Features in Genetic Absence Epilepsy and in Acutely Induced Absence Seizures

**DOI:** 10.1155/2013/875834

**Published:** 2013-05-07

**Authors:** A. S. Bazyan, G. van Luijtelaar

**Affiliations:** ^1^Institute of Higher Nervous Activity and Neurophysiology, Russian Academy of Science, Russian Federation, 5A Butlerov Street, Moscow 117485, Russia; ^2^Biological Psychology, Donders Centre for Cognition, Donders Institute for Brain, Cognition and Behavior, Radboud University Nijmegen, P.O. Box 9104, 6500 HE Nijmegen, The Netherlands

## Abstract

The absence epilepsy typical electroencephalographic pattern of sharp spikes and slow waves (SWDs) is considered to be due to an interaction of an initiation site in the cortex and a resonant circuit in the thalamus. The hyperpolarization-activated cyclic nucleotide-gated cationic *I*_h_ pacemaker channels (HCN) play an important role in the enhanced cortical excitability. The role of thalamic HCN in SWD occurrence is less clear. Absence epilepsy in the WAG/Rij strain is accompanied by deficiency of the activity of dopaminergic system, which weakens the formation of an emotional positive state, causes depression-like symptoms, and counteracts learning and memory processes. It also enhances GABA_A_ receptor activity in the striatum, globus pallidus, and reticular thalamic nucleus, causing a rise of SWD activity in the cortico-thalamo-cortical networks. One of the reasons for the occurrence of absences is that several genes coding of GABA_A_ receptors are mutated. The question arises: what the role of DA receptors is. Two mechanisms that cause an infringement of the function of DA receptors in this genetic absence epilepsy model are proposed.

## 1. Introduction

**Figure 1:**
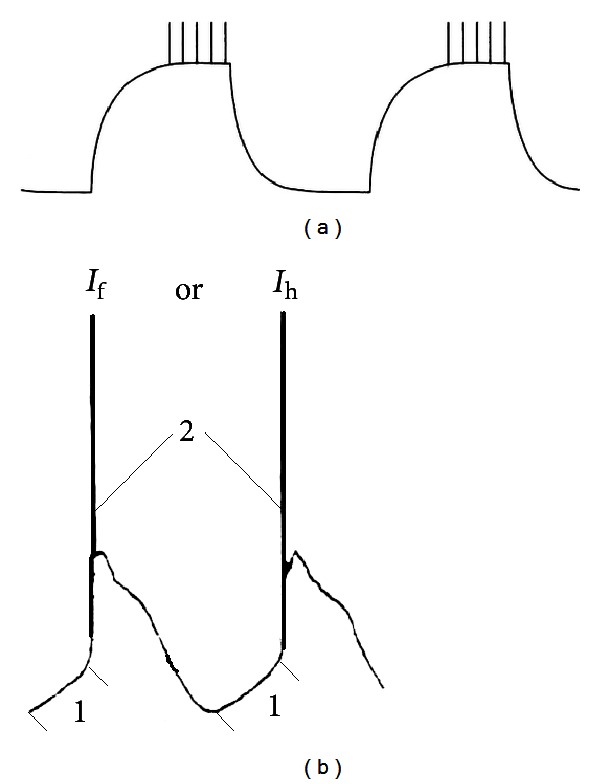
The schematic representation of two types of epileptic discharges. (a) The gradual increase in potential reaches the threshold level followed by the generation of epileptic discharges. This is typical for the EEG hallmarks accompanying convulsive seizures. (b) The spike-wave discharge. It consists of an inhibitory phase and an action potential. The inhibitory phase (1) appears as a slow wave on the EEG, excitation (a series of action potentials) of the cells appears as a spike (2). A rebound peak appears at the end of this phase and the cycle repeats itself many times. The discharge possesses the same profile as spike-wave discharges (with permission from Springer; [30]).

Absence seizures, typical for many different patients with absence epilepsy, principally differ from convulsive seizures. To illustrate, a number of widely used and efficient anticonvulsant drugs enhance absence seizures in patients and in the genetic absence models and ethosuximide is only effective in suppressing absence seizures and completely ineffective in other types of seizures [1–3]. Absence epilepsy is characterized by the occurrence of spontaneous occurring spike-wave discharges (SWDs) and periods of reduced alertness or responsiveness. The SWDs are induced by hyperpolarization and they appear on an otherwise normal EEG. The spike of the SWD represents an excitatory postsynaptic potential and bursts of action potentials (Figure 1(b)) of thalamocortical and corticothalamic cells, the wave a slow wave in the EEG, is the subsequent inhibitory phase [4, 5]. The generalized and widespread bilaterally synchronous SWDs are the result of highly synchronized oscillations in corticothalamocortical networks. SWDs, as can be found in WAG/Rij rats, have a local cortical origin in the perioral region of the somatosensory cortex [6–12], which is now confirmed in GAERS [13, 14], another well validated and often used strain of rats with absence epilepsy [15, 16]. Local injections in the perioral region of the somatosensory cortex of the T-type Ca^2+^ channel blocker ethosuximide was rather effective in suppressing SWDs [17], but also in Long-Evans rats with SWDs, and in Wistar rats treated with a low dose of PTZ [18]. The cortex is part of a network in which the reticular thalamic nucleus (RTN) is an essential part. The RTN is responsible for the initiation of sleep spindles not only in rats, but also in other species [19, 20]. It needs to be acknowledged that previously the RTN was considered as the pacemaker for SWDs as well [21, 22]. Nowadays the origin of the initiation site of SWDs is the subject of quite some research efforts, in rats as well as in humans and it is widely assumed that the cortex contains a focal hyperexcitable region in which SWDs have their origin, although the initiation site and the number of foci might be different among patients and between humans and rats [10, 14, 23–29].

The genetic rodent models are most widely used as models of absence epilepsy. They allow the investigation of the neurobiological underpinning of this type of epilepsy and the accompanying SWDs [9, 16, 31–36]. The mice have often spontaneous mutations such as in lethargic and tottering mice and transgenic and gene replacement models are also often used (for review see [35, 36]). These mice have spontaneous epileptic seizures of which the behavioral and electrographic features and anticonvulsant sensitivity might sometimes be similar to those of human absence seizures [2]. Acutely induced absence seizures can also be studied in acute pharmacological models, *γ*-hydroxybutyrate (GHB), penicillin but most often a low dose of pentylenetetrazole (PTZ) is used [37]. There are two pharmacological models for atypical absence epilepsy. These are the AY-9944 [38, 39] and the methylazoxymethanol acetate (MAM)-AY-9944 [40] models in rats. Administration of the 7-dehydrocholesterol reductase inhibitor, AY9944, early in life to either rats or mice results in the spontaneous recurrent occurrence of atypical absence seizures which last throughout the animal's lifetime. The MAM-AY9944 model is a double hit model of medically refractory atypical absence epilepsy since cortical dysplasias are first induced in rat by prenatal administration of MAM and then AY9944 is administered during the first 3 weeks postnatally [41].

The WAG/Rij model is one of the well documented models [9, 10, 16, 34, 42]. The SWDs of WAG/Rij rats start to appear in the EEG when the rats are about 2-3 months. At the age of six months, all rats of this fully inbred strain have several hundred SWDs per day. The model has some features, which further increase the interest.

- WAG/Rij have a changed expression of genes coding for the low threshold T-type Ca^2+^ channel (*I*_Ca,T_) compared to ACI control rats [43] and absence epileptic patients have a mutation of genes coding for *I*_Ca,T_ [44–46].
- Reduction of expression of the *α*3 subunit protein in GABA_A_ receptors in the RTN [47] of WAG/Rij rats and mutation of genes coding for subunits of GABA_A_ receptors in people with absence epilepsy were found [48, 49].
- WAG/Rij rats have complex phenotype: they are comorbid for depression [50–52].

We assume that the *I*_h_ pacemaker channels localized in the somatosensory cortex contribute to the initiation of the occurrence of SWDs and that a second set of pacemaker channels can be found in the thalamus. The latter is responsible for the occurrence of thalamic delta activity and sleep spindles, as can be assumed from the classical studies of the firing pattern in thalamic relay cells, tonic and burst firing modes [53, 54]. A small portion of thalamic relay cells show bursting during SWDs, in which *I*_h_ might also play a role. The thalamus, a recipient of many cortical efferents, is also part of a resonant circuitry for SWDs that can be activated by the somatosensory cortex. In this way, the RTN and its widespread connections to other thalamic nuclei form a pathway in the propagation of cortically triggered SWDs and sustain oscillations.

## 2. Hyperpolarization-Activated *I*_h_ Pacemaker Channel in the Mammalian Brain

The pacemaker channels in the mammalian brain are named *I*_h_ channels, the pacemaker channels in other tissue are named *I*_f_ [30, 55]. Both are cationic pacemaker channels.

### 2.1. The Features of *I*_f_ and *I*_h_ Pacemaker Channels

**Figure 2:**
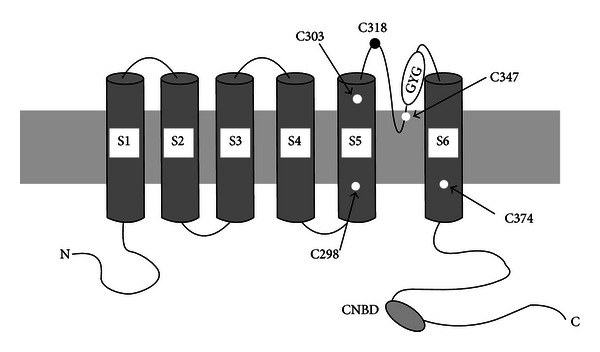
Probable transmembrane topology of HCN channels. Six transmembrane domains (S1–S6) of monomeric of the HCN1 subunit are presented. Five endogenous cysteine residues are localized in transmembrane domains of all four isoforms of HCN channels. Three of them in the 303, 318, and 347 position (Cys303, Cys318, and Cys347) are localized closer to the extracellular space, whereas the two remaining ones (Cys298 and Cys374) are probably closer to the cytoplasmic or inner side. The cyclic nucleotide-binding domain (CNBD) is localized on the C-terminus. The glycine-tyrosine-glycine amino-acid sequence forms a channel pore between the S5 and S6 domains (with permission from Springer, [30]).

Hyperpolarization-activated cyclic nucleotide-gated cationic *I*_f_ or *I*_h_ pacemaker channels maintain spontaneous periodic activation, and they were discovered in heart (*I*_f_) and brain (*I*_h_). Four isoforms of this channel are known (HCN1–HCN4, hyperpolarization-activated and cyclic nucleotide-gated) [56–61]. HCN channels resemble voltage-dependent K^+^ channels; they are tetrameric and consist of monomeric subunits, each of which contains six transmembrane domains (S1–S6) with a P-loop (Figure 2) between the S5 and S6 domains [60, 62]. Four HCN1 subunits join and form a HCN1 homotetrameric channel, four HCN2 subunits form a HCN2 homotetrameric channel, and so forth. Different HCN subunits may also join to form a heteromultimeric complex, this substantially increases the diversity of *I*_f_, or *I*_h_ channels [62–64].

The primary analysis of the HCN channel sequence [56–58, 60, 62] indicated the presence of five endogenous cysteine residues in the transmembrane domains. Three of these are localized closer to the extracellular side, the Cys303 residue in the S5 transmembrane domain, the Cys318 residue in the aminoacid chain connecting S5 and S6, and Cys347, which is in direct proximity to the glycine-tyrosine-glycine (GYG) sequence that forms the channel pore. Two other cysteine residues are localized closer to the cytoplasmic, or inner, side (Cys298 in S5 and Cys374 in S6) (Figure 2). It is known that cysteine has a hydrosulphate group and its reduced form may easily participate in chemical reactions, like alkylation, acylation, and arylation, or even cross-inking with another nearby cysteine to form a disulphide bridge [65, 66]. These chemical reactions are essential for association of subunits and channel formation.

The HCN channel is open at an average membrane potential of −80 mV [67]. However, different subunits of the HCN channel possess different functional properties. To illustrate, HCN1 and HCN2 subunit isoforms, coexpressed in the cerebral cortex and hippocampus, are substantially different in their biophysical features. HCN1 channels become five to ten times faster activated than HCN2 channels. Also, HCN1 channels become activated at a membrane potential that is 20 mV more positive than the potential required for HCN2 activation [68, 69]. The HCN2 channel demonstrates a clear response (+17 mV) to cAMP binding at the cyclic nucleotide binding domain (CNBD) on the C-terminus, the HCN1 channel demonstrates a minimal response to cAMP (+4 mV). Therefore it can be safely assumed that HCN1 and HCN2 perform quite different functions in the brain regions where they predominate [70]. Heteromultimeric channels with new properties can be found throughout the central nervous system [68, 69]. Coexpression of HCN1 and HCN2 with heteromultimeric channel formation leads to *I*_h_ currents, which are kinetically activated via a voltage-dependent mechanism and tend to possess functional properties that are intermediate between HCN1 and HCN2 homomeric channels. Coexpressed heteromultimeric channels demonstrate a relatively larger shift in response to cAMP (+14 mV) than the monomeric HCN1 channel. Neither the kinetic curve nor dose-effect curve in response to cAMP of coexpressed heteromultimeric *I*_h_ channels are a linear summation of the independent populations of HCN1 and HCN2 homomeric channels. These heteromeric channels have unique and new properties [63].

Several chimers of HCN1 and HCN2 homomeric channels have been created with a modified NH_2_ terminus, transmembrane domain, and different areas of the COOH terminus. CNBD exchange between HCN1 and HCN2 results in only a small influence on the main gating mechanism, and exerts a relatively poor effect on cAMP modulation. The difference in the cAMP modulatory effect depends instead on the interaction between CNBD and the aminoacid sequence (80 amino acids) that connects the last transmembrane domain (S6) and CNBD.

The glycine-tyrosine-glycine (GYG) sequence found between the S5 and S6 domains forms the channel pore (Figure 2). The pore is formed by this aminoacid sequence originating from all four subunits when they are united in the tetrameric channel. The same amino acid sequence forms a pore in many K^+^ selective channels [62]. The pore of the HCN channel is permeable not only for K^+^ but also for Na^+^ ions.

### 2.2. *I*_h_ Channels and Absence Epilepsy

The main diagnostic sign of absence epilepsy is the presence of SWDs in human or animal electroencephalogram (EEG). The similarity in morphology of SWDs [32, 71] and discharges generated by *I*_h_ channel (Figure 1(b)) suggests that the *I*_h_ channel is a contributing factor to the morphology of the spike and wave complex. Several other authors have already hypothesized that this nonselective *I*_h_ channel plays also a crucial role in the age-dependent development in the occurrence in SWDs [70, 72–76]. Considering that different subtypes are differently present in cortex and thalamus, HCN1 is expressed selectively in specific brain regions, among others the neocortex and certainly on the large pyramidal neurons [58, 77, 78], while HCN2 is more widely expressed throughout the brain, including the thalamus; these different parts will be discussed separately.

#### 2.2.1. *I*_h_ in Cortex

Studies aimed at elucidating the role of *I*_h_ channel activity in absence epilepsy compared *I*_h_ channels in cortical neurons of epileptic and control strains of rats: WAG/Rij, Wistar (of the same genetic background as WAG/Rij but less prone to absence epilepsy) and ACI rats (an inbred strain practically devoid of epileptic activity) [73, 79, 80]. The cortex was targeted since it contains the focal onset zone of SWDs. Patch-clamp recording from the whole cell of the second-third cortical layer of pyramidal neurons was supplemented by immunohistochemical, Western blot, and PCR studies of HCN1–HCN4 subunits of the *I*_h_ channel. The fast component of *I*_h_ activation in neurons of WAG/Rij rats was significantly reduced (a 50% decrease in the current density) and was four time slower than in the neurons of nonepileptic Wistar or ACI rats. The results of Western blot and PCR analysis corresponded to a decreased *I*_h_ current. A decrease by 34% was found in the level of the HCN1 subunit protein in the cerebral cortex of WAG/Rij rats as compared to Wistar rats, but HCN1 mRNA expression was not different. The protein and mRNA levels of the other three *I*_h_ channel subunits (HCN2–HCN4) were not altered [73]. This loss of neocortical HCN1 function may contribute to an increased cortical excitability since there are substantially fewer HCN1 subunits in the combined complex of the *I*_h_ channel in the cortical zone containing the focal region in WAG/Rij strain compared to the two control strains.

A loss of HCN1 was also found during ontogeny: it occurs primarily in the apical dendrites of layer 5 pyramidal neurons in the cortex, leading to a spatially uniform 2-fold reduction in dendritic HCN current throughout the entire somatodendritic axis [76]. Dual whole-cell recordings from the soma and apical dendrites demonstrate that loss of HCN1 increases somatodendritic coupling and significantly reduces the frequency threshold for generation of dendritic Ca^2+^ spikes by back propagating action potentials. As a result of increased dendritic Ca^2+^ electrogenesis, a large population of WAG/Rij layer 5 neurons showed intrinsic high-frequency burst firing. Using morphologically realistic models of layer 5 pyramidal neurons from control Wistar and WAG/Rij animals, it was shown that the experimentally observed loss of dendritic *I*_h_ recruits dendritic Ca^2+^ channels to amplify action potential-triggered dendritic Ca^2+^ spikes and increase burst firing. Thus, loss of function of dendritic HCN1 channels in layer 5 pyramidal neurons provides a somatodendritic mechanism for increasing the synchronization of cortical output, and is therefore likely to play an important role in the generation of SWDs accompanying absence seizures [76].

It has been shown that neonatal handling and maternal deprivation postnatally (1–21 days) resulted in reduced number of absences, decreased interspike interval, and changes in power of the frequency spectrum of SWDs in adult WAG/Rij rats [81]. Whole cell patch-clamp recordings from the cells of the fifth pyramidal layer, in situ hybridization, and Western blot analysis of the cortex of adult WAG/Rij rats [74] showed an increase in the HCN1 protein level in the somatosensory cortex of handled and mother-deprived rats as compared to control rats. This increase was selective for the HCN1 subunit and did not affect the expression of HCN2–HCN4 subunit proteins; neither did expression of the mRNA of any subunit (HCN2, HCN3, and HCN4). These results indicate that relatively mild changes in the environment of neonatal rats have long-lasting consequences for the occurrence of paroxysmal activity later in life and suggest that increased concentration or better, an increased proportion of HCN1 subunits in *I*_h_ channel composition in a small area of the somatosensory cortex is related to a reduction in the number of SWDs. An increased number of HCN1 subunits in the channel due to their extensive expression (increased HCN1 protein level without changes in HCN2, HCN3, and HCN4 mRNA levels), and consequently, a decreased number of HCN2, HCN3, and HCN4 subunits in the channel complex, as mentioned above [68, 69], result in impaired activity of the *I*_h_ channel, reduced cortical excitability, and less SWDs, as it has been demonstrated experimentally [73, 74, 81].

Besides the age-dependent decrease in the number of HCN1 subunits and in protein expression in the deep layers of the focal region [76], a chronic drug treatment aimed at antiepileptogenesis in the same model confirmed that the reduction of SWDs was accompanied by an increase in HCN1, producing levels that resemble nonepileptic controls [82]. In addition, neonatal manipulations [74] increased the number of HCN1 subunits and decreased SWDs, providing evidence for the negative correlation between SWDs on the one side and this *I*_h_ channel otherwise.

Blocking the *I*_h_ channels of Layer V neurons by ZD7288 (4-ethylphenylamino-1,2-dimethyl-6-methyl-aminopyrimidinium chloride), as well as a loss of HCN1 subunits, increases somatodendritic coupling, increases Ca^2+^ conductance by reducing the frequency threshold for induction of dendritic Ca^2+^ electrogenesis, increasing synchronization of cortical output and burst firing [76]. Therefore, ZD7288 when administered in the cortex can enhance SWDs. It is, however, possible that this property of *I*_h_ channels is only present in neurons of the the subgranular layers, since *I*_h_ channels of thalamic neurons have different biophysical properties.

It can be concluded that an age-dependent loss of neocortical HCN1 function contributes to an increased cortical excitability in the form of more and longer spontaneous occurring SWDs and that various early manipulations, such as maternal deprivation but also treatments aiming at antiepileptogenesis confirm the negative relation between HCN1 and SWDs. 

#### 2.2.2. *I*_h_ in Thalamus: Its Role in Delta, Sleep Spindles, and SWDs

The in vitro studies done in the late eighties and in the nineties of the last century were influential for the ideas than have been developed on the role of the interaction between *I*_h_ and *I*_Ca,T_ [83]. Basically, the interaction of *I*_h_ and *I*_Ca,T_ channels has been assumed to be the basis for burst firing in thalamocortical (TC) cells [84]. Briefly, upon hyperpolarization of the TC cell membrane by a reduction of depolarizing inputs beneath the resting membrane potential, *I*_h_ is activated and produces a slow pacemaker depolarization until the threshold voltage of activation of *I*_t_ is reached. The hyperpolarization concomitantly deinactivated *I*_t_ and the pacemaker depolarization subsequently activates *I*_t_ that leads to the generation of a low-threshold Ca^2+^ potential, bringing the membrane potential positive to firing threshold. This evokes high-frequency burst of action potentials. The transient nature of *I*_t_ will then terminate the depolarization and hyperpolarize the cell. Less *I*_h_ (less inward current) than at the beginning of the Ca^2+^ spike will be activated because *I*_h_ partly deactivated during the depolarization associated with the Ca^2+^ potential. As a consequence, the membrane potential will hyperpolarize to a potential more negative than the *I*_t_ activation threshold. The cell will thus reach the peak of the hyperpolarization and reactivate *I*_h_, restarting the cycle. Thus, while *I*_t_ is responsible for the large-amplitude depolarizations, *I*_h_ mediates both the slow pacemaker depolarization necessary to activate *I*_t_ and the hyperpolarization that follows low-threshold Ca^2+^ potentials [85]. The burst firing mode is activated during drowsiness, with TC and CT cells undergoing a progressive hyperpolarization from drowsiness and the early stages of sleep to the late sleep stage of deep slow wave sleep. The frequency of the burst firing mode is determined by the amount of hyperpolarization, such that sleep spindle oscillations (7–12 Hz) occur at more depolarized membrane potentials than delta oscillations [86]. It has been assumed based on in vitro studies that this thalamic burst firing mode can also be the physiological correlate of SWD. However, studies in GAERS have demonstrated that TC relay cells were not associated with rhythmic IPSPs, Ca^2+^ currents, or burst discharges, but only with the occasional firing of a single action potential [87, 88], and that during SWDs a minor portion of TCR cells (7% in GAERS, [87]) show Ca^2+^ currents and burst discharges. The bursting of such a small portion of TCR cells appears to be sufficient for the sustainment of SWDs. In agreement with this finding is that local injections of the low-threshold Ca^2+^ blocker ethosuximide are not very active when injected in the thalamus [17]. Moreover, there are no in vivo studies in genetic models which investigated the role of thalamic *I*_h_.

However, burst firing in TC cells is crucial for the presence of delta waves. In in vitro rat, cat, and ferret studies, isolation of TC relay cells from synaptic input from CT and RTN leads to a hyperpolarization of the cells and onset of self-sustained oscillations in the delta (0.5–4 Hz) frequency band [85, 89, 90]. Likewise hyperpolarization of TC neurons by means of hyperpolarizing current injection leads to burst firing of the neurons also in the delta frequencies [85], and this goes together with the activation of *I*_h_ [85, 89]. In vivo studies which confirmed this were, however, not done.

Studies on the role of *I*_h_ in spindle oscillations have been performed in thalamic slices with neurons that either show spontaneous spindling, such as in the ferret LGNd [91], or where thalamic spindle activity is evoked by applying hyperpolarizing current pulses mimicking IPSPs arriving during spindle waves in the intact RTN/PGN-TC network [92]. Induced spindle activity in TC neurons has been shown to go together with *I*_h_ activation, lending evidence for an involvement of *I*_h_ in spindle generation [93, 94]. Further evidence for a role of *I*_h_ in spindle oscillations comes from *I*_h_ manipulation studies in thalamic slices. Intracellular injection of depolarizing current into spindling LGNd neurons resulted in inactivation of *I*_t_, abolition of rebound Ca^2+^ spikes and cessation of the spindle oscillations [95]. The type of burst firing oscillation (delta and spindle oscillations) is determined by the membrane potential [86], that is on its turn set by *I*_h_ [70, 93]. *I*_h_ thus plays an important role in setting the membrane potential that determines the type of burst firing oscillation present in the TC cells. But different from delta oscillations, *I*_h_ and the pacemaker potential created by low-threshold Ca^2+^, does not seem to be of critical importance for spindles since blockade of *I*_h_ does not lead to the cessation of the oscillation, as seen in delta oscillations [85, 89, 96, 97], but to a failure in spindle termination [89, 92]. This questions the exact role of *I*_h_ in spindle generation; moreover in vivo studies towards the role of *I*_h_ in sleep spindle generation are lacking as well.

It can be concluded that the interaction of *I*_h_ and *I*_t_ seems critical for the generation of delta oscillations, *I*_h_ seems involved in sleep spindle generation, and however its exact mechanisms are beyond the scope of this paper. A dominant role for *I*_h_ in thalamic bursting underlying SWD can also be doubted since a small portion of TC cells show this behavior. However, the possibility exists that *I*_h_ and *I*_t_ interact in the portion of TC cells that do show bursting in which the *I*_h_ and *I*_t_ interact as a tandem.

#### 2.2.3. *I*_h_ in Thalamus: A Different Role

The role of thalamic *I*_h_ was investigated in WAG/Rij rats: high HCN1 mRNA and protein expression without alterations in HCN2–HCN4 expression was found in thalamic neurons [98]. These authors found also that that burst discharges in thalamocortical neurons locked to SWDs were prolonged during blockade of *I*_h_ by Cs^+^ and ZD7288, similar as has been found for sleep spindles. This was associated with hyperpolarizing shift in *I*_h_ activation, a decrease in cAMP responsiveness of *I*_h_ in TC neurons and resulting impairment to shift from burst to tonic firing, which in turn produced long burst activity after recruitment of *I*_h_ during SWDs. On the basis of these results and shifts of *I*_h_ channel reactivity induced by high concentrations of cAMP, Budde et al. [98] hypothesized that increased HCN1 expression in the epileptic thalamus is associated with decreased *I*_h_ channel reactivity to cAMP in thalamic neurons and leads to a loss of control over the channel, which, in turn, prolongs high *I*_h_ channel activity during absence seizures.

Interesting results [75] were also obtained during the study of the regulation of the pacemaker *I*_h_ channel in the thalamus of GAERS rats; the function of the *I*_h_ channel and subunit composition in TC-neurons were studied before (presymptomatic) and when (symptomatic) SWDs were present in the EEG. The voltage-dependent *I*_h_ current remained unaltered in mature somatosensory neurons, both in vivo and in vitro. However, an increase in *I*_h_ current by the phasic, practically physiologic, impulse of cAMP was diminished by approximately 40% and the half-maximal cAMP concentration was approximately fivefold increased. This diminished sensitivity of *I*_h_ current to its main cellular modulator preceded the beginning of epilepsy, lasted during its chronic phase, and accompanied the elevated expression of mRNA of the cAMP-insensitive HCN1 subunit of the channel (>50%) without alterations in HCN2–HCN4 mRNA expression.

Changes in excitability of TC neurons were analyzed by measuring the delayed upregulation of the *I*_h_ current. This current was caused by Ca^2+^-triggered cAMP synthesis and is essential for finishing synchronized oscillations in vitro. Repeated recovery of the Ca^2+^ spike induced the normal deceleration of upregulation of the *I*_h_ current in mature GAERS neurons, sufficient to reduce the spontaneous rhythmic burst discharges. These adaptive mechanisms take place despite cAMP turnover and involve increased intracellular Ca^2+^ concentration in response to repeated low-threshold Ca^2+^ discharges. Thus, HCN channels may play an ambient role in absence epilepsy. The weakening of the binding of cAMP to HCN channels in the thalamus proceeds, and likely promotes, epileptogenesis in GAERS rats, whereas compensatory mechanisms stabilizing the function of *I*_h_ channels contribute to the completion of the SWDs in chronic absence epilepsy [75].

**Figure 3:**
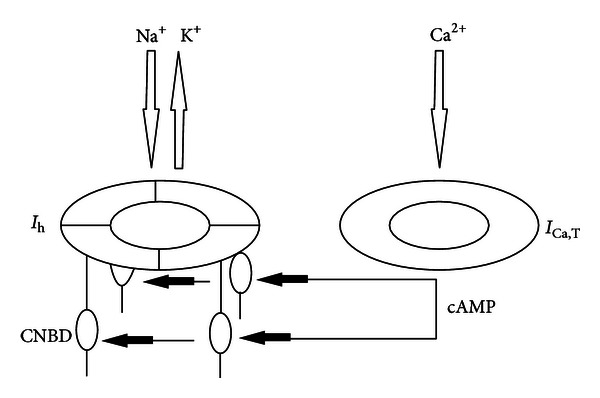
Schematic representation of the functional interaction between *I*_h_ and *I*_Ca,T_ channel, for explanation see text.

A quantitative model (Figure 3) describes how *I*_h_ and *I*_t_ cooperate and how the subcompositions of the subunits determine the net effect (depolarization) of hyperpolarizing inputs. It is based on Kuisle et al. [75]. The tandem consisting of *I*_h_ and *I*_Ca,T_ works as follows: the hyperpolarization of the cell membrane opens a *I*_h_ channel and the membrane is depolarized by an amount *N*. Next the hyperpolarization opens the *I*_Ca,T_ channel and Ca^2+^ enters the cell, increases activity of adenylate cyclase, and increases cAMP concentration as the result of selective activation of PKA (cAMP-dependent protein kinase-A) isoforms. The cAMP binds to the CNBD domain and enhances the ion flow and amount of membrane depolarization (gating mechanism) to a value *M*. The total amount of depolarization *I*_h Dep_ = *N* + *M*.

When the *I*_h_ channel consists of four HCN1 subunits, as the case in the cortex for the large pyramidal cells, then the interaction of cAMP with the CNBD HCN1 subunit strengthens the gating mechanism and the depolarization is increased by 4 mV [63]. In this case, 4(HCN1); *I*_h Dep_ = *N* + (4 × 4) mV = *N* + 16 mV.

When cAMP interacts with CNBD of an HCN2 subunit, as is the case in the thalamus, then the gating mechanism is amplified and the depolarization is increased by 17 mV [63]. The subunit composition can be either:

  3(HCN1) + 1(HCN2); *I*_h Dep_ = *N* + (3 × 4) mV + (1 × 17) mV = *N* + 29 mV
  2(HCN1) + 2(HCN2); *I*_h Dep_ = *N* + (2 × 4) mV + (2 × 17) mV = *N* + 42 mV
  1(HCN1) + 3(HCN2); *I*_h Dep_ = *N* + (1 × 4) mV + (3 × 17) mV = *N* + 55 mV
  4(HCN2); *I*_h Dep_ = *N* + (4 × 17) mV = *N* + 68 mV.

This implies that the composition of the heteromeric *I*_h_ channel, as can be found for example, in the thalamus, determines the amount of depolarization and the subsequent activation of voltage-activated Ca^2+^ channels. With age, the contribution of HCN1 subunits decreases (through a process called trafficking) and is replaced by HCN2 subunits. It does also explain that during aging the reaction to hyperpolarizing inputs might increase the amount of depolarization; in other words, during aging the thalamus will react with larger depolarizations upon the same hyperpolarizing inputs. 

Our hypothesis that burst firing in TC neurons is caused by (tandem) hyperpolarization-activated *I*_h_ channel and hyperpolarization-activated (*I*_Ca,T_) channel and that burst firing of at least some TC cells plays a role in SWD occurrence, is supported by pharmacological studies indicating that increased GABAergic inhibition in the ventral basal (VB) complex of the thalamus, as induced by local injections of GABA-agonists, enhances SWDs. Remember that opposite effects (a decrease in SWDs) were obtained by injections of GABA agonists in the focal zone of the cortex [15, 99, 100], suggesting that here depolarization rather than hyperpolarization promotes burst firing [76, 101].

The literature reviewed in this paragraph suggests that *I*_h_ and low-threshold T-type Ca^2+^ channels in TC cells work in tandem in order to generate burst-like activity, the assumed neurophysiological firing pattern that forms the basis of delta waves, sleep spindles and perhaps SWDs. The latter remains uncertain since the vast majority of TC cells do not show burst firing activity during SWDs. Hyperpolarization opens the *I*_h_ channel, and cationic current depolarizes the membrane to the threshold and induces a Ca^2+^ spike. Hyperpolarization also opens the *I*_Ca,T_ channels. The entrance of Ca^2+^ ions into the cell induces Ca^2+^-dependent cAMP synthesis, and cAMP dramatically increases channel activity through binding to the CNBD locus of HCN subunits. The HCN1 subunit responds weakly to cAMP binding; therefore, a decrease in the proportion of HCN1 subunits in the channel increases pacemaker activity and an increase in the proportion of HCN1 subunits in the channel decreases pacemaker activity. The analysis of experimental data shows that one of the basic mechanisms of the long-term regulation of *I*_h_ pacemaker activity is modification and consolidation of a number of HCN1 subunits in the pacemaker channel by the help of the transduction signal.

## 3. GABAergic Inhibitory System

### 3.1. Pharmacological Investigations

The GABAergic system has two types of receptors. The first type of receptors mediating fast postsynaptic GABA reactions (IPSP's) are ionotropic GABA_A_ receptors, including in its structure a chlorine channel. The second type of receptors mediating slow GABA reactions is metabotropic GABA_B_ receptors. GABA_B_ receptors are coupled indirectly to K^+^ channels. When activated, these receptors can decrease Ca^2+^ conductance and inhibit cAMP production via intracellular mechanisms mediated by G proteins. GABA_B_ receptors can mediate both pre- and postsynaptic inhibition. Presynaptic inhibition may occur as a result of GABA_B_ receptor binding on nerve terminals causing a decrease in the influx of Ca^2+^, thereby reducing the release of neurotransmitters. Postsynapticaly, the G protein-gated inwardly rectifying K^+^ (GIRK) channels are activated by stimulation of G_i/o_-coupled GABA_B_ receptors and provide an important mechanism for the slow inhibitory modulation of cellular excitability in the brain [102–104].

#### 3.1.1. GABA_A_ Receptors

The GABA_A_ receptors can be allosterically facilitated (increase of chlorine current) by benzodiazepines [105–107]. Pharmacological studies with specific agonists and antagonists of the benzodiazepine (BDZ) receptor focused on the GABA_A_ receptor complex. A series of drugs belonging to the class of the *β*-carbolines were evaluated, mainly partial agonists such as ZK 91296 and ZK 112119 (abecarnil) and compared with the full agonist diazepam [108–110]. Diazepam showed all behavioral and electrophysiological changes characteristic for the BDZ, ZK 91296 reduced seizure activity without inducing signs of sedation, sleepiness, myorelaxation, and changes in behavior or EEG spectral content. Another member of the *β*-carboline family, the partial inverse agonist FG 7142, aggravated epileptic activity, with slightly enhanced immobile behavior, suggesting anxiogenic properties. The results not only demonstrate that the multiple effects of the BDZ could be separated by these compounds, but also that the antiepileptic activity is not related to changes in spectral content of the EEG. Because of its selective activity, ZK 91296 appears to be more suitable than diazepam in reducing seizure activity. Finally, FG 7142 seems a genuine partial inverse agonist which has some, but not all, of the inverse effects of a full agonist [108]. Next the effects of the *β*-carboline (abecarnil, ZK 112119) were determined in two experiments: in the first the antiabsence profile of abecarnil was evaluated in WAG/Rij rats. In the second experiment, effects on sleep and behavior were investigated in nonepileptic Wistar rats. It was found that, similarly to classical BDZ, abecarnil possessed a strong antiepileptic character and also changed the background EEG to more high-frequency waves and less spindle activity. It also produced more immobile behavior. Abecarnil induced only small, marginally significant increases in slow-wave sleep while reducing REM sleep as a proportion of total sleep. It also reduced the number of REM periods. These observations are consistent with the proposed partial agonist activity of abecarnil, a drug with interesting therapeutic implications [111]. Rather similar results on the antiabsence action of the full agonists of BDZ receptors have been found in GAERS [112], emphasizing the similarities in the pharmacological profile of the two genetic absence models.

Hypnotic drugs are known to possess antiepileptic activity. Therefore, the effects of the BDZ hypnotic midazolam and the imidazopyridine hypnotic Zolpidem (both 10 mg/kg) on sleep-wake states and on the number of SWDs were evaluated in WAG/Rij rats. Both hypnotics reduced the number and duration of SWDs. The initial decrease of SWDs after midazolam, however, was followed by a rebound. The antiabsence activity of zolpidem mimics that of midazolam and is well correlated with the equipotent hypnotic action and anticonvulsant effect in the isoniazid test [113].

The assumed tolerance to BDZ was investigated by injecting diazepam (5 mg/kg) twice daily for 23 days in WAG/Rij rats. After this, the rats received the agonist, diazepam, or the antagonist, flumazenil (Ro 15-1788). Acutely administered, diazepam reduced SWDs, while flumazenil was without clear effects. The effects were rather different after chronic administration: both compounds increased the number of SWDs [114], suggesting that repeated administration of diazepam abolished its antiepileptic action and even reversed its action. Also Flumazenil produced an increase in SWDs after chronic diazepam treatment. The antiepileptic effect of diazepam, acutely administrated, has been found also by others in GAERS [115].

**Figure 4:**
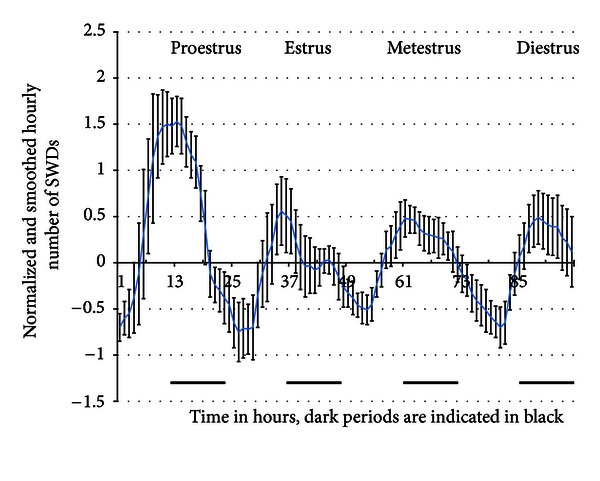
Hourly number of SWDs (mean and sem) in female WAG/Rij rats across the ovarian cycle. Note the circadian pattern across all four days and the increase in SWDs at the last hours of the light and first hours of the dark period of the proestrus day. It is at specifically these hours that progesterone levels are increased (adapted, [121]).

The next allosteric facilitator of GABA inhibition at the GABA_A_ receptor is the group of neurosteroids [116]. They have clinical relevance partly because the ovarian hormones acting as neurosteroids are held responsible for the variation of seizure frequency during the menstrual cycle, around puberty, and menopause. The variations in seizure frequency have been attributed partly to the antiepileptic action of progesterone: it reduces seizures during the luteal phase. However, studies on catamenial epilepsy (derived from the Greek word *katomenios*, meaning “monthly”), clusters of seizures around specific points in the menstrual cycle, have not always distinguished between patients with focal and generalized seizures. Grünewald et al. [117] described a patient whose absence seizures exacerbated when she was given progesterone. This indicates that, in contrast to the well-known anticonvulsant and antiepileptic properties of progesterone in most animal models and in almost all seizure types, absence epilepsy might be an exception in the sense that progesterone increases absence seizures [118]. The role of the sex hormones, but also of ganaxolone and other neurosteroids, has been thoroughly investigated in the genetic and pharmacological absence models. Ganaxolone (3*α*-hydroxy-3*β*-methyl-5*α*-pregnan-20-one) is a synthetic neurosteroid with anticonvulsant properties in a number of seizure models as well as the ability to enhance function of the GABA_A_ receptor complex via the neurosteroid binding site. The effect of ganaxolone on absence seizures was assessed in the low-dose PTZ and the GHB model. Ganaxolone resulted in a significant prolongation of absence seizure in both models and in doses above 20 mg/kg it produced bilaterally synchronous SWDs associated with behavioral arrest. These data suggest that augmentation of GABA_A_ receptor complex function by neurosteroids has the potential to result in or exacerbate absence seizures [119]. Systemic administration of the neurosteroids allopregnanolone and pregnenolone sulfate was studied in WAG/Rij rats. Allopregnanolone is also a positive modulator of the GABA_A_ receptor, it increased dose dependently the number and total duration of SWDs. Pregnenolone sulfate, also a positive modulator of NMDA receptors, also increased those parameters, though only at the highest dose used (100 mg/kg) and only in the first hour post-injection. The obtained data indicate that both these neurosteroids aggravate SWD activity. These proepileptic activities of these neurosteroids contrasts with the anticonvulsant effects of many of these neurosteroids and they point to a different pharmacological profile of epilepsy with convulsive or nonconvulsive seizures [120]. In the next experiment the influence of endogenous neurosteroids on SWDs was investigated in female WAG/Rij rats in a chronic EEG study during a complete ovarian cycle [121]. A circadian pattern emerged for the number of SWDs: a nadir during the first hours of the light period, and an acrophase during the first hours of the dark period. This daily maximum was increased at proestrus day compared with the other days of the cycle, coinciding with an increased progesterone plasma level specifically at these hours of this day. This clearly suggests that even shifts in physiological concentrations of progesterone are already large enough to enhance SWDs. The results are presented in Figure 4.

There was no difference in the first few hours of the light period in the number of SWDs between proestrus and the three other days, suggesting that estradiol has no effect on SWDs. In the second study, the effects of systemic administration of progesterone and 17*β*-estradiol on SWDs and spontaneous behavior were investigated. It was confirmed what was found in the long term EEG study: progesterone increased the number and total duration of SWDs; 17*β*-estradiol was completely ineffective. The administration of RU 38486, an antagonist of intracellular progesterone receptors, had no effect on SWDs and did not block the stimulatory effect of progesterone. The intracellular estrogen receptor antagonist tamoxifen did not evoke alterations in the number or duration of SWDs either. The results indicate that estradiol is not affecting SWDs and that progesterone aggravates SWDs, but the progesterone effect is not mediated through intracellular receptors. Since progesterone is rapidly metabolized in the brain to the positive modulator of GABA_A_ receptor allopregnanolone, which is also known to increase SWDs in WAG/Rij rats, it is rather likely that the epileptoformic effects of progesterone are mediated through this metabolite [121]. In order to elucidate whether the regulatory effect of progesterone depends indeed on its conversion to allopregnanolone, the effect of finasteride, a 5*α*-reductase inhibitor, on progesterone-induced increase in SWDs was studied. Progesterone dose dependently increased the number of SWDs during the first hour after injection. Finasteride alone had no effect on the number of SWD, up to 24 h following its administration. Pretreatment of rats with finasteride, however, blocked the progesterone-induced enhancement of SWD. This indicates that the action of progesterone is mediated by its neuroactive metabolite allopregnanolone, through strengthening of GABA_A_ receptor activity [122].

The administration of both progesterone and allopregnanolone exacerbated seizures in the AY model (an atypical absence epilepsy model) in agreement with the outcomes in WAG/Rij rats, whereas 17*β*-estradiol attenuated SWDs (this ovarian hormone does not modulate absences in the typical absence model). Neither mifepristone nor tamoxifen blocked the effects of progesterone and 17*β*-estradiol, respectively, on SWDs duration in the atypical absence model, suggesting that these two sex hormones are working in a manner independent of their intracellular neurosteroid receptor [123]. Anyway, the differential role of estradiol in typical and atypical absence seizures emphasizes the differences between these types of epilepsy [41]. 

The effects of neurosteroids on SWDs in WAG/Rij rats were also studied by local microinjections [99]. Allopregnanolone and ganaxolone (the two positive allosteric modulators of the GABA_A_ receptor complex acting on the steroid recognition site) and pregnenolone sulphate, (the negative allosteric modulator of GABA_A_ receptors and a positive modulator of NMDA receptors) were investigated. Focal bilateral microinjection of the two GABA_A_ positive modulators into some thalamic nuclei (nucleus ventralis posteromedialis, NRT, nucleus ventralis posterolateralis) increased SWDs. Both compounds reduced number and duration of SWDs when microinjected into the perioral region of the primary somatosensory cortex, the focal region. This demonstrates that there is not a general increase in inhibition, instead there seems to be an increased inhibition on the level of the VB, not a decrease in inhibition in the RTN [15] and the focal zone in the cortex. The effects of pregnenolone sulphate were more complex and depended on the dose and the site of administration: generally, at low doses in RTN and focal cortical region an increase in number and mean duration was found, and a reduction at high doses. The effects in the ventralis posteromedialis and posterolateralis were a decrease and no effect, respectively, on SWDs. It was concluded that neurosteroids might play a role in absence epilepsies and that their action depends on the activity in specific neuronal areas. More specifically, the results show that a lack of inhibition at the focal cortical site may be a cause of the increased excitability.

Some researchers have studied the role of the GABA_A_ receptor site. The effects of specific GABA_A_ receptor agonist and antagonist, muscimol and bicuculline, respectively, were studied by intracerebroventricular injections. EEG registrations and behavioral observations showed that muscimol dose dependently increased the nonconvulsive absence seizures. Besides this, it induced EEG spikes and body twitches. Bicuculline induced spikes and body twitches as well but decreased the nonconvulsive epilepsy. All effects of muscimol were blocked by bicuculline and vice versa, which suggests that the observed effects are genuine GABA_A_ effects [124]. The other agonists of GABA_A_ receptors such as 4,5,6,7-tetrahydroisooxazolo(5,4-c)pyridin-3-ol (THIP), L-baclofen [115, 125] enhanced the duration of SWDs in a dose-dependent fashion.

All these described results implicate that nonconvulsive epilepsy can be caused by a GABA_A_ receptor hyperfunction, although this is an oversimplification. In general, the opposite action of GABAmimetic drugs points to a pharmacological difference between convulsive and absence types of epilepsy. The local injection studies demonstrated that there is GABA_A_ hyperfunction in the VB, but a GABA_A_ hypofunction in the focal cortical region.

#### 3.1.2. GABA_B_ Receptor

The GABA_B_ receptor antagonist CGP 35348 was intraperitoneally given in doses of 100, 300, and 900 mg/kg to old Wistar rats, endowed with similar SWDs as WAG/Rij rats and GAERS. The effects on sleep and behavior were investigated as well. The low and middle dose of the drug produced an increase in the duration of REM sleep, the REM sleep latency was correspondingly reduced. Non-REM sleep and total sleep duration increased and an s-shaped dose-response relationship was found. Explorative behavior was diminished. Number and duration of SWDs were reduced after all doses of CGP 35348, confirming the strong suppressive action of this GABA_B_ antagonist on absences [126]. Others have described similar effects in GAERS [127] and in the lethargic (lh/lh) mutant mice model [128]. Antagonists of the GABA_B_ receptors suppressed SWDs whereas agonists of GABA_B_ receptors exacerbated them. Furthermore, GABA_B_ receptor binding and synaptically evoked GABA_B_ receptor-mediated inhibitions of NMDA responses were selectively increased in lh/lh mice. Therefore, enhanced GABA_B_ receptor-mediated synaptic responses may underlie absence seizures in the lh/lh mice, and GABA_B_ receptor antagonists hold promise as antiabsence drugs [128]. The microinjections of the GABA_B_ agonist [(−)-baclofen] and the antagonist CGP 35348 in anterior ventral lateral thalamic nucleus, RTN and nucleus reunions decrease and increase, respectively, absence seizures in (lh/lh) mice [129]. Next the antiabsence effect of the GABA_B_ receptor antagonist SCH 50911 was investigated in the lethargic (lh/lh) mutant mouse and in two rat models in which absence seizures were induced by administration of either GHB or a low-dose (20 mg/kg) PTZ. SCH 50911 abolished seizures in all three models in a dose-dependent fashion. In each model SCH 50911 was more potent than the GABA_B_ receptor antagonist CGP 35348, ethosuximide, trimethadione, and valproic acid. SCH 50911 was equipotent as the GABA_B_ antagonist CGP 46381 in the lh/lh mouse model [130]. Further experiments examined the effectiveness of a range of specific GABA_B_-receptor agonists and antagonists of varying specificity, as well as the specific GHB-receptor antagonist NCS 382, in the GHB and low-dose PTZ models. All specific GABA_B_-receptor antagonists as well as the specific GHB-receptor antagonist produced blockade of absence seizures in both models; pretreatment with GABA_B_ receptor agonists resulted in generalized absence status epilepticus lasting for hours. These data confirm that specific GABA_B_-receptor antagonists have antiabsence activity, that the same holds for specific GHB-receptor antagonists, and raise the possibility that both GHB- and GABA_B_-antagonist drugs have the potential to be useful therapeutic agents in generalized absence seizures [131].

Systemic injections of the GABA_B_ receptor antagonists suppress completely SWDs in GAERS [127]. In the same strain, bilateral injections of the selective GABA_B_ receptor agonist R-baclofen into the specific relay nuclei and the RTN increased SWDs dose dependently, whereas injections of a GABA_B_ antagonist CGP 35348 into the same sites decreased these seizures dose dependently.

The effect of R-baclofen on SWDs could be blocked by a subsequent injection of CGP 35348 at the same site. Injections of R-baclofen or CGP 35348 in the midline thalamus had no effect. Bilateral injections of R-baclofen into the specific relay nuclei of nonepileptic rats induced synchronized rhythmic oscillations on the cortical EEG. These results suggest that GABA_B_ receptors in the ventrolateral thalamus and in the RTN are involved in an oscillatory activity which underlies SWDs [132]. SWDs in GAERS were also dose dependently suppressed by i.p. administration of the GABA_B_ receptor antagonists CGP 36742 and CGP 56999, and by bilateral microinjections of the same compounds into the lateral nuclei of the thalamus. In rats susceptible to audiogenic seizures, intraperitoneal administration of both GABA_B_ receptor antagonists, at doses which suppressed absence seizures, facilitated the elicitation of sound-induced tonic seizures. In nonepileptic control rats, intraperitoneal injections of higher doses of CGP 36742 and CGP 56999 induced delayed clonic convulsions, which were suppressed by pretreatment with baclofen. c-Fos protein was expressed after GABA_B_ receptor antagonist-induced clonic seizures in the cortex, hippocampus, amygdala, perirhinal, and piriform cortex. Intracortical and hippocampal microinfusion of both GABA_B_ receptor antagonists produced focal seizures. The authors concluded that GABA_B_ receptor antagonists induce convulsions in cortical and limbic structures but suppress nonconvulsive absence seizures by blocking thalamic GABA_B_ receptors [133]. 

Somewhat different conclusions were drawn by Staak and Pape [22] in WAG/Rij rats: their in vivo studies in anesthetized WAG/Rij rats in combination with single-unit recordings and microiontophoretic techniques in the ventrobasal thalamic complex showed that the application of the GABA_B_ receptor antagonist CGP 55845A exerted a statistically insignificant modulatory effect on neuronal activity during spontaneous SWDs but significantly attenuated the bicuculline-evoked aggravation of SWD-related firing. This indicates that GABA_A_ receptor-mediated events are recruited with each SWD and that SWD-related activity can be evoked with no significant contribution of GABA_B_ receptors. It is obvious that their results voted against a predominant or even exclusive contribution of GABA_B_ receptors to spontaneous SWDs in thalamic relay nuclei in the WAG/Rij strain, but rather point to a critical role of GABA_A_ receptor activation. Whether this difference is due to the neurolept anaesthetic is not immediately clear.

The GABA_B_ receptor antagonists CGP55845A and CGP62349 suppressed the development of the absence syndrome to a larger extent in mice than in rats with GHBL-induced absence epilepsy in a chronic treatment protocol in which both GHBL and the GABA_B_ antagonists were given for 33 days. The absence syndrome was observed after 3 weeks of treatment in the saline group. A third antagonists CGP71982 suppressed it later than the other two antagonists (fifth week) [134], suggesting that GABA_B_ receptors are also involved in epileptogenesis.

**Figure 5:**
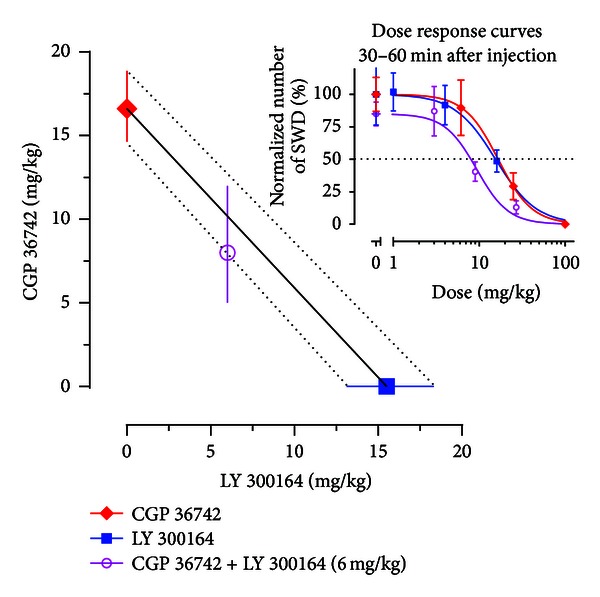
Isobologram for the antiabsence interaction between the GABA_B_ antagonist CGP 36742 and the AMPA receptor antagonist LY 300164. The ED_50_ value of CGP 36742 is plotted on the ordinate (closed diamond) and that of LY 300164 on the abscissa (closed square). The ED_50_ values (for the inhibition of SWD) were obtained by fitting data to the sigmoid *E*_max⁡_ model, for the data from the time period 30–60-min after injection (see graph insert and Materials and Methods section of [135]). The straight line connecting the two plotted ED_50_ values is the isobolographic line, while dotted lines represent 95% confidence intervals (CIs). The experimental ED_50_ of the combination of CGP 36742 and LY 300164 (6 mg/kg) is plotted as the open circle and 95% CIs as the solid vertical line. If the experimentally determined ED_50_ lies on the isobolographic line, then the drug effects are additive. If the ED_50_ lies below this line, supra-additivity is assumed and when the ED_50_ lies above the isobolographic line, there is infra-additivity. However, when 95% CIs of the experimentally obtained combination overlap with the respective intervals of the isobolographic line, then the interaction is regarded as statistically nonsignificant. In such case, the observed interaction seems to be additive (reprinted with permission from Elsevier, [135]).

The effects of combined and single administration of the GABA_B_ receptor antagonist CGP 36742 and *α*-amino-3-hydroxy-5-methyl-4-isoxazolepropionic acid (AMPA) receptor antagonist, 7,8-methylenedioxy-1-(4-aminophenyl)-4-methyl-3-acetyl-4,5-dihydro-2,3-benzodiazepine (LY 300164) on SWDs were also investigated in WAG/Rij rats in order whether the combined treatment might yield additive effects. CGP 36742 was effective as it reduced the number and mean duration of SWDs dose dependently. LY 300164 had minor effects: only the highest dose (16 mg/kg) reduced the number of SWDs in a short time window. The ED(50) values for the inhibition of SWDs by LY 300164 and CGP 36742 in a time window 30–60 min after injection were 15.5 and 16.6 mg/kg, respectively. The ED(50) of CGP 36742 was reduced to 8.0 mg/kg when this antagonist was administered in combination with LY 300164 (6 mg/kg). The interaction between the two antagonists appeared to be additive according to isobolographic analysis. The data are presented in Figure 5.

Importantly, CGP 36742 and LY 300164 administered either alone or in combination had no apparent effects on behavior. Their isobolographic method used may provide information for a rational approach to polytherapy in general and specifically for the treatment of generalized absence epilepsy [135].

A similar effect for specific antagonist of GABA_B_ receptor has been received in the AY-9944 model of atypical absence epilepsy [136]. The GABA_B_ receptor antagonist CGP 35348, administered i.p., decreased dose dependently the mean burst times. SWDs were suppressed for at least 4 h after injection of CGP 35348.

#### 3.1.3. Physiological Inactivation of GABA

There are two mechanisms of the physiological inactivation of GABA: high affinity reuptake of GABA by specific GABA transporters and enzymatic inactivation of GABA by GABA-transaminase. The inhibition of both mechanisms of GABA inactivation by specific agents leads to a substantial increase of GABA concentration in the synaptic cleft.

In a series of clinical reports the development of nonconvulsive status epilepticus (NCSE) with electroclinical features consistent with those of atypical absence seizures after adjunctive antiepileptic therapy of tiagabine (an inhibitor of GABA reuptake) was described. The patient, a boy of 7 years, suffered from frequent myoclonic seizures and rare atypical absences, tonic, atonic, and simple partial seizures of unknown etiology. The interictal EEG showed generalized sharp waves and/or 2–2.5 Hz. NCSE developed when rapid dosage increase and high dose of tiagabine was given [137]. In two other reports, it was described that tiagabine induced NCSE in patients with focal epilepsies and in one patient with juvenile myoclonic epilepsy. A 32-year-old patient with absence epilepsy and primary generalized tonic-clonic seizures since 11 years of age was described, who developed her first NCSE while treated with 45 mg of tiagabine daily [138]. Three other cases with focal lesional epilepsy had nonconvulsive status epilepticus induced by treatment with tiagabine [139].

These clinical cases were predicted based on results in the genetic absence models: effects of tiagabine in various doses were investigated on EEG, two types of SWDs, and behavior of WAG/Rij rats. WAG/Rij rats have two types of SWDs, the most common ones, bilateral symmetrical and generalized (type I), and more occipital, shorter lasting SWDs (type II). According to expectations from other GABAmimetics, Tiagabine enhanced in a dose-related way both the number and mean duration of both types of SWDs. The low dose of 1 mg/kg had almost no effects, but doses of 3 and 10 mg/kg were effective. Furthermore, tiagabine in the latter two doses increased the power in the higher *β* band of the background EEG, whereas no significant changes in behavior of the rats were found. In general, this experiment supports that nonconvulsive epilepsy is associated with a GABA hyperfunction. It also underlines the biochemical differences of convulsive and nonconvulsive animal models of epilepsy since tiagabine is rather effective in blocking convulsive seizures; it belongs to the category of drugs effective in convulsive animal models and not in nonconvulsive models of epilepsy [1]. 

The limbic system is generally not included in any theory (for review see [7]) about the pathogenesis of absence seizures. However, some data demonstrated that the alterations in the limbic system attribute to the expression of absence epileptic phenotype in genetic models of absence epilepsy [140]. Tolmacheva and van Luijtelaar [141] investigated whether local intrahippocampal administration of the neurosteroid progesterone and the GABA reuptake inhibitor tiagabine might affect the occurrence of SWDs. WAG/Rij rats received intracerebral injections of progesterone, 45%  *β*-cyclodextrin (CD), saline, or tiagabine. EEG recordings made before and after injection showed that progesterone, CD, and tiagabine administration to the hippocampus reduced SWDs for 60 min following administration without behavioral or electroencephalographic side-effects. Both progesterone administration into the cortex and saline injection into the hippocampus yielded no changes in the occurrence of SWDs. These data suggest that activation of GABAergic transmission in the hippocampus has an inhibitory effect on corticothalamocortical circuits underlying the generation of SWDs and might be critically involved in the regulation of absence seizures in the corticothalamocortical network. 

Tiagabine increases absence seizures also in the other genetic absence models such as after systemic administration in GAERS and in the lethargic mouse models [2, 142].

The intensification of the efficiency of the GABAergic system by vigabatrin, a GABA-transaminase inhibitor, causes also an increase of absences. Vigabatrin but also carbamazepine and phenytoin are contraindicated in typical absence seizures [143]. Yang et al. [144] presented three patients with de novo absence epilepsy after administration of carbamazepine and vigabatrin with different types of epilepsy. In a second clinical study 14 patients (seven male), aged 15–46 years, and with a mean duration of epilepsy of 16.4 years were identified. Video-EEG demonstrated typical absence status epilepticus in five, atypical status epilepticus in five, atypical myoclonic status epilepticus in three and typical myoclonic status epilepticus in one. Epilepsy had been misclassified as cryptogenic partial in eight cases and cryptogenic generalized in four. The correct diagnosis proved to be juvenile absence epilepsy in six patients, juvenile myoclonic epilepsy in four, epilepsy with generalized tonic-clonic seizures on awakening in two, and childhood absence epilepsy in two. All patients had been treated with carbamazepine (CBZ) and had experienced seizure aggravation or new seizure types before referral. Seven patients had polytherapy with phenytoin, vigabatrin, or gabapentin. Potential precipitating factors included dose increase of carbamazepine or of carbamazepine and phenytoin; initiation of carbamazepine, vigabatrin, or gabapentin; decrease of phenobarbital. Withdrawal of the aggravating agents and adjustment of medication resulted in full seizure control. This series shows that severe pharmacodynamic aggravation of seizures in idiopathic generalized epilepsy may result in absence status epilepticus or myoclonic status epilepticus, often with atypical features [145].

The effects of vigabatrin on type II SWDs in the EEG of ACI rats were studied in order to learn more about the effects of altering GABA concentration on SWDs. The incidence and mean duration of type II SWDs increased after vigabatrin as compared to saline. This effect appeared with a halftime of 100 min. The peak frequency of the type II SWDs decreased after vigabatrin (5.6 Hz) as compared to saline treatment (7.5 Hz). Thus, vigabatrin alters the type II SWD morphology. These results are in agreement with predictions of Destexhe's theoretical model, modulating both GABA_A_ and GABA_B_ conductance's [146]. The effect of vigabatrin on the starting and the stopping mechanisms (the latter was determined by the hazard function) was also investigated in WAG/Rij rats. This experiment showed that a high GABA level changed the stopping mechanism of the absence epileptic seizures, creating much better conditions for very long seizures to occur. With respect to the starting mechanism, it was found that both with a high and a low GABA level, there was evidence for a recovery mechanism that decreases the probability that a new seizure starts. Another possibility is that the brain protects itself from a subsequent absence seizure, an endogenous protection mechanism; this has been described for the hippocampus by Kelly and McIntyre [147] and for acute PTZ seizures [148]. Initially this probability is lower with high GABA concentrations, but gradually it converges to the same constant baseline probability as in the condition with a low GABA concentration [149].

Other inhibitors of GABA catabolism such as *γ*-vinyl GABA (GVG) also increase SWDs, as demonstrated in GAERS [115, 125, 150].

Thus, pharmacological research of GABA_A_ and GABA_B_ receptors and of GABA inactivation mechanisms specifies the basic strengthened role of the GABAergic system on absence seizures. Such conclusion can be made on the basis of that in the overwhelming majority agonists of GABA receptors after systemic injections increase SWD activity (an exception are the benzodiazepines), and antagonists reduce them. Besides strengthening of GABAergic neurotransmission, a more exact prolongation of GABA effects by inhibition of GABA inactivation mechanisms also increases absences. Local injections of GABA mimetic drugs may show opposite effects, so that one can conclude that absence seizure is not due to a global increase in inhibition, but to localized increased and decreased sites of inhibition. The increase of SWDs by GABA agonists confirms our assumption that the hyperpolarization-induced *I*_h_ pacemaker in the thalamus contributes to the occurrence of SWDs. The composition of the *I*_h_ channel isoforms in the focal region of the cortex, the putative presence of *I*_h_ on GABA interneurons in different part of the brain, together with the found opposite actions of GABA agonists in cortex and thalamus, might give room for another collaboration between *I*_h_ and (*I*_Ca,T_).

### 3.2. Measurement of GABAergic System Efficiency

One of the methods to estimate the efficiency of neurotransmitter systems is to determine the binding constants of a labeled ligand with a receptor; it characterizes the activity of a receptor. By the help of specific concentration-dependent kinetic reactions and equations describing them, the dissociation constant (*K*_d_) and density of a receptor (*B*_max⁡_) type are defined. Rate of direct and reverse reactions of interaction (i.e., rate of association and dissociation) depends on the concentration of the ligand. The dissociation constant (*K*_d_) is the concentration ligand at which the rate of direct and reverse reactions is equal, then the rate of association is equal to the rate of dissociation. The *K*_d_ defines the affinity of a ligand with a receptor. The general pool of receptors in a membrane can be in two states: closed state (receptors immerse in the cell membrane, with such type of receptors binding is not carried out) and in open state, when receptor binding takes place. An increase of receptor density (*B*_max⁡_) implies an increase in the quantity of the active centers of binding. Based on *K*_d_ and *B*_max⁡_, it is possible to characterize receptors. A reduction of *K*_d_ (without changes in *B*_max⁡_) means increase in affinity of receptors, strengthening of efficiency of the reaction of the receptors to the most sensitive and intensive part of a receptor. An increase of *B*_max⁡_ (without change in *K*_d_) means strengthening of receptors reaction efficiency to all concentrations tested. 

In vitro, the binding parameters of ^3^H-Ro 5-4864, a ligand labeling the peripheral benzodiazepine receptor, were determined for brain homogenates of WAG/Rij and ACI rats. No difference in *K*_d_, but a significant decrease (25%) in WAG/Rij rats receptors *B*_max⁡_, was found [151]. Autoradiography was used to study the binding of [^3^H]-GABA with GABA_A_ and GABA_B_ receptors in brains from GAERS: both the density of GABA_A_ or GABA_B_ receptors and affinity of these receptors to GABA were determined. No difference in the density of GABA_A_ and GABA_B_ receptors and affinity of these receptors to GABA between control and epileptic animals were found [152]. The regional distribution of radioactive ligand binding for different receptors of the GABA_A_-benzodiazepine-picrotoxin chloride channel complex was measured on tissue section by autoradiography in brains taken from GAERS and controls. The ligands employed included [^3^H]muscimol for high affinity GABA agonists sites; [^3^H]SR 95531 for the low-affinity GABA sites; [^3^H]flunitrazepam for the benzodiazepine sites; and [^35^S]t-butyl bicyclophosphorothionate (TBPS) for the picrotoxin site. There were no significant differences between GAERS and control animals in [^3^H]flunitrazepam and [^35^S]TBPS binding. However, there was a decreased [^3^H]muscimol and [^3^H]SR 95531 *B*_max⁡_ in the CA2 region of the hippocampus in GAERS. It is necessary to note once more that the hippocampus is traditionally not considered as a structure in which SWDs can be measured [4, 21, 153], although recent data in GAERS and WAG/Rij rats point towards alternations in the limbic system as a consequence of the hundreds daily SWDs [41, 140]. Another study explored a possible role of GABA_B_ receptors in absence seizures in lethargic (lh/lh) mice. [^3^H]baclofen binding to neocortical plasma membranes prepared from lh/lh and wild (+/+) age-matched congenic mice was measured. The *B*_max⁡_ of GABA_B_ receptors was 20% larger in lh/lh than in +/+ mice in an age-independent manner. Interestingly, the subset of lh/lh mice with larger seizure frequency (40–70 seizures/15 min) had a significantly greater *B*_max⁡_ than the subset with lower seizure frequency (1–10 seizures/15 min). The increase of GABA_B_ receptor density was selective, because binding to glutamate receptors ([^3^H]glutamate binding) and to GABA_A_ receptors ([^3^H]muscimol binding) was not significantly different in the two strains. The authors suggest that higher density of GABA_B_ receptors in lh/lh mice underlies the expression of absence seizures in this model [154]. GABA and the enzyme of its synthesis, glutamic acid decarboxylase (GAD), was investigated in cortex and thalamus of GAERS by immunocytochemistry; the GABA_A_ receptors were evaluated by autoradiography of ^3^H-flunitrazepam binding and by immunocytochemistry using specific antibodies against the *β*2-*β*3 subunits of GABA_A_ receptor protein. GABA and GAD immunocytochemistry did not show any difference in density or distribution of immunoreactive elements (fibers, terminals and neurons) between epileptic and control animals, but autoradiographic and immunocytochemical studies showed a decreased enhancement of ^3^H-flunitrazepam binding and of *β*2-*β*3 subunits of GABA_A_ receptor in the sensory-motor cortex and anterior thalamic areas of the epileptic strain. No differences were found in benzodiazepine receptors in the two strains. GABA_B_ receptors were measured as ^3^H-baclofen binding in a crude synaptic membrane preparation and there was no difference between epileptic and control animals. The data suggest an impairment of the “GABA_A_ system” in studied brain regions of epileptic rats, due to a reduction of receptor *β*2-*β*3 subunits and coupling to benzodiazepine receptors despite the normal synthesis and location of the neurotransmitter [155]. The effect of GHBA-induced SWDs on the function of various components of the GABA_A_ receptor complex in the cortex of the rat was determined in a series of in vitro experiments. GHBA itself without SWDs had no effect on the binding of [^3^H]muscimol, [^3^H]flunitrazepam, and [^35^S]TBPS or on the uptake of  ^36^Cl^−^ into synaptoneurosomes in the in vitro studies. However, at the onset of GHBA-induced SWDs, there was a significant decrease in the binding of [^35^S]TBPS, associated with a significant decrease in muscimol-stimulated uptake of ^36^Cl^−^ with no other biochemical change. One minute after onset of GHBA-induced absence seizures, a significant increase in the binding of [^3^H]muscimol was noted. Ten minutes later the decrease in muscimol-stimulated uptake of ^36^Cl^−^ had normalized, while the changes in binding of [^3^H]muscimol and [^35^S]TBPS persisted [156]. The analysis of extra- and intracellularly recorded synaptic responses revealed an intracortical hyperexcitability which was accompanied by a significant reduction in the efficiency of GABAergic inhibition. These data indicate that the imbalance between intracortical excitatory and inhibitory mechanisms may at least contribute to the expression and augmentation of SWDs in WAG/Rij rats, and this was also found in an in vitro neurophysiologic study [157]. Leaning on the known fact, that postsynaptic GABA_B_ receptor-mediated events are reduced after prior treatment with pertussis toxin (PTx), the influence of PTx (0.4 microg), denatured-PTx or vehicle saline bilaterally injected into the relay nuclei of the thalamus in anesthetized GAERS were studied for up to 6 days, following which the brains were removed and GABA_B_ or GABA_A_ receptor autoradiography was performed. By 6 days the SWD of the rats treated with PTx was suppressed by 96% compared with vehicle-injected rats with a significant (62%) reduction even after 1 day. Denatured toxin had no effect. After 6 days GABA_B_, but not GABA_A_, receptor binding was significantly reduced by 70–80% in the ventrolateral and ventral posterolateral thalamic nuclei [158]. In the same model, the high affinity binding of GABA_A_/BZD receptors with (^3^H)RO 15-1788 in the brain of naive rats and after administration of FG 7142 did not differ in GAERS and nonepileptic control rats [112]. The authors suggested that the hypersensitivity of GAERS to various inverse agonists of the GABA_A_/BDZ receptor involves cortical GABA_A_ receptors and is not related to differential activity of a subunit-selective receptor.

Miniature GABA_A_ IPSCs recorded in the VB complex of the thalamus, and in cortical layer II/III neurons were similar in GAERS and nonepileptic controls, whereas in GAERS RTN neurons had a 25% larger amplitude and 40% faster decay. In addition, baclofen was significantly less effective in decreasing the frequency of RTN mIPSCs in GAERS than in control, whereas no difference was observed for cortical and VB mIPSCs between the two strains. Paired-pulse depression was 45% smaller in GAERS RTN, but not in VB, and was insensitive to GABA_B_ antagonists. These results point to subtle, nucleus-specific, GABA_A_ receptor abnormalities underlying SWDs rather than a full block of these receptors across the whole corticothalamocortical network. Their occurrence prior to seizure onset suggests that they might be critically involved in epileptogenesis [160]. 

**Figure 6:**
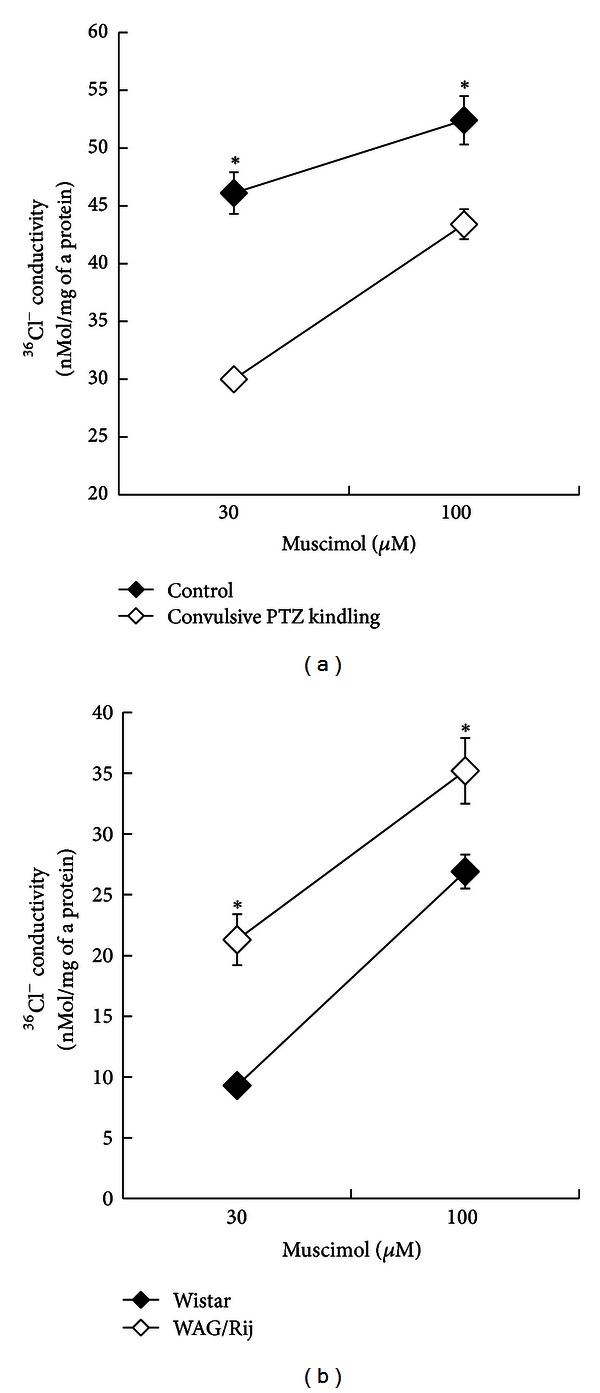
Muscimol-induced ^36^Cl^−^ conductivity (nmole/mg of protein) inside synaptoneurosomes prepared from the neocortex of 3-4-month-old control Wistar rats (a) and Wistar rats with PTZ-induced convulsive kindling and 5-6-month-old control Wistar and WAG/Rij rats (b). *P < 0.05 (with permission from Springer, [159]).

We have studied muscimol-induced ^36^Cl^−^ conductivity in synaptoneurosomes prepared from the frontal and somatosensory cortex of rats with three types of epileptic activity: tonic-clonic pentylenetetrazole kindling in Wistar rats, nonconvulsive absence PTZ kindling in Wistar rats (an absence seizure model), WAG/Rij and Wistar control rats [159]. We used two concentrations of muscimol: 30 and 100 *μ*M. The occurrence of kindling prior to tonic-clonic seizures in the Wistar rats decreased the muscimol-induced ^36^Cl^−^ conductivity compared to control (Figure 6(a)). Development of nonconvulsive kindling considerably increased ^36^Cl^−^ conductance into the neocortical synaptoneurosomes. WAG/Rij rats showed an increase in ^36^Cl^−^ conductance in neocortical synaptoneurosomes as compared to the control Wistar rats (Figure 6(b)). The decrease in muscimol-induced ^36^Cl^−^ conductivity after development of tonic-clonic kindling was in agreement with a quite some amount of data regarding the decrease in the activity of GABA_A_ receptor during tonic-clonic kindling [55, 161–165]. The high level of muscimol-induced ^36^Cl^−^ conductivity in the neocortical synaptoneurosomes of the WAG/Rij rats supported the concept that the SWDs originating from the somatosensory cortex are induced by hyperpolarization. The high level of ^36^Cl^−^ conductivity during nonconvulsive PTZ kindling suggests that the activity of the GABA_A_ receptor was similar in the genetic absence epilepsy model and PTZ absence seizure model.

The binding of [^11^C]flumazenil at the BDZ site of the GABA_A_ receptor was studied in five patients with idiopathic generalized epilepsy with positron emission tomography. No evidence was found for a change in [^11^C]flumazenil binding with absence seizures [166]. However, these early studies were not aimed at the cortex, the most likely location of the origin of SWDs.

Except for binding constants of labeled ligands with GABA_A_ and GABA_B_ receptors defining efficiency of GABAergic, other parameters defining efficiency of GABAergic transmission have been used. Levels of extracellular GABA and other amino acids in the ventrolateral thalamus in GAERS have been monitored with in vivo microdialysis. It was shown that the basal extracellular levels of GABA and, to a lesser extent, taurine were increased when compared with values in nonepileptic controls. However, modifying GABAergic transmission with the GABA_B_ agonist (−)-baclofen, the GABA_B_ antagonist CGP-35348, or the GABA uptake inhibitor tiagabine did not produce any further alteration in extracellular GABA levels [167]. Another study was performed to test the hypothesis that presynaptic GABA_B_ receptors in lh/lh mice inhibit [^3^H]GABA release to a greater degree than nonepileptic littermates (designated +/+). Synaptosomes isolated from neocortex and thalamus of age-matched lh/lh and +/+ mice were similar in uptake of [^3^H]GABA. In the neocortical preparation, baclofen dose dependently inhibited [^3^H]GABA release evoked by 12 mM KCl, an effect mediated by GABA_B_ receptors. The maximal inhibition (*I*_max⁡_) value was significantly greater (80%) in lh/lh than in +/+ mice, whereas the IC50 (3 microM) was unchanged. The effect of baclofen (50 microM) was 58% less robust in thalamic preparation of the lh/lh mice. Other effects mediated by GABA_B_ receptors (inhibitions in Ca^2+^ uptake and cAMP formation) were also significantly reduced in thalamic synaptosomes from lh/lh mice. It is possible that selective effects of presynaptic GABA_B_ receptors or GABA release in neocortex and thalamic nuclei of lh/lh mice may contribute to mechanisms underlying absence seizures [168]. K^+^ conductance of GABA_B_ receptor activation by baclofen was subsequently studied. Whole-cell voltage-clamp recordings were made from thalamic ventrobasal neurons of age-matched lethargic (lh/lh) and wildtype (+/+) mice. No essential distinctions were found [169]. GABA release and uptake were examined in GAERS and in nonepileptic control animals, using crude synaptosomes prepared from the cerebral cortex and thalamus. Uptake of [^3^H]GABA over time was reduced in thalamic synaptosomes from epileptic rats, compared to controls. The affinity of the uptake process in thalamic synaptosomes was lower in epileptic animals. NNC-711, a ligand for the GAT-1 uptake protein, reduced synaptosomal uptake by more than 95%; *β*-alanine, an inhibitor selective for the uptake proteins GAT-2 and GAT-3, did not significantly reduce synaptosomal uptake. These results indicate that GABA uptake in the thalamus of GAERS rats was reduced compared to control animals. The lower level of uptake in the epileptic animals corresponds to higher efficiency of GABA system in these rats [170]. 

Progesterone and estradiol serum levels were investigated in WAG/Rij rats before, during, and after pregnancy following parturition. The serum concentration of progesterone increased after the 3rd day of pregnancy. Levels of estradiol changed less. Progesterone is kept high until the 18th day of pregnancy and drastically decreased before the parturition. Common duration of absences-spontaneous SWD, frequency, and the duration of every SWD decreased from 3rd to 19th days of pregnancy before parturition. On the basis of these data and regulation of GABA_A_ receptor efficiency by neurosteroids [116], it can be assumed that the changes in the parameters of SWD are possibly correlated with changes in progesterone in serum during pregnancy [171, 172]. 

A time course study that examined the effects of the female estrous cycle on the more slow atypical SWDs, GABA_B_ receptor binding, and GABA_B_ receptor protein expression was conducted in the AY9944 model in Long-Evans rats. There was a significant increase in both the duration of SWDs and GABA_B_ receptor binding during the proestrus stage of the estrus cycle, the stage during which the levels of progesterone are at their highest. No changes in GABA_B_R1 or R2 protein levels were observed. These data suggest an important role for steroid hormones and its interaction with GABA receptors in the regulation and maintenance of AY9944-induced atypical absence seizures [123]. Brain cholesterol synthesis inhibition (CSI) at a young age in rats has been shown to be a faithful model of the symptomatic generalized epilepsies, in this case acquired absence epilepsy, a devastating condition for which few therapies or models exist. The CSI model was used to study cellular mechanisms. Patch-clamp and whole-cell recordings were compared from neurons acutely dissociated from the RTN in Long-Evans Hooded rats treated and untreated with the cholesterol synthesis inhibitor U18666A. In U18666A-treated animals, 91% of rats developed SWDs. Relevant is also that mutations of the *γ*2 subunit of the GABA_A_ receptor abolish natural sensitivity to BDZ [173]. Patch-clamp results revealed that although there was no remarkable change in GABA_A_ receptor affinity, both a loss of ability of benzodiazepines to enhance GABA_A_-receptor responses and an increase of Zn^2+^ inhibition of GABA_A_-receptor responses of RTN neurons occurred in this model. This change was specific, since no significant changes were found in neurons exposed to the GABA allosteric modulator pentobarbital. Taken collectively, these findings provide evidence for abnormalities in BDZ and Zn^2+^ modulation of GABA_A_ receptors in the cholesterol synthesis inhibition model and suggest that decreased *γ*2 subunit expression may underlie important aspects of generation of TCSWDs in atypical absence seizures. The present results are also consistent with recent findings that mutation of the *γ*2 subunit of the GABA_A_ receptor changes benzodiazepine modulation in families with generalized epilepsy syndromes [174]. 

It is known that basket cells typically express two calcium-binding proteins, parvalbumin (PV) and calbindin. PV is co-expressed with GABA in 90% of the GABAergic neurons [175, 176], and PV-immunostaining is an appropriate way to mark basket cells [177]. Quantification of PV-positive cells showed clear reductions in the parietal and forelimb area of the somatosensory cortex in WAG/Rij rats compared to nonepileptic control ACI rats. These results are interpreted as deficiency of Ca^2+^-binding protein in corresponding structures of the brain of WAG/Rij rats [178], or as deficiency of GABAergic cells [9].

Thus, the measurement of efficiency of the GABAergic system in various absence models leads to the conclusion that a huge variety of experiments demonstrate a large role for deficiencies in the GABAergic system. However, there are also diverse results, among others with the role of GABA_B_. We see three reasons for this; different seizure or epilepsy models were used. It is possible that different deficiencies and different mechanisms may all lead to the same phenotype: SWDs. The second reason of the diverse results is the so-called process of uncertainty of the nervous system [179]. The analysis of several works has shown that increase or decrease of general activity of the neurotransmission system at different animals can be carried out by different ways and depends on specific features of these animals. The third reason for the occurrence of diverse results can be processes of indemnification (protection against injury). To illustrate, in our study of synaptoneurosomes, samples from cortex of Wistar and WAG/Rij rats were leveled on protein [159] and on concentration of GABA_A_ receptors. In these conditions conductivity of chlorine was higher in WAG/Rij rats. In brain tissue, in which GABA systems have been reduced [9], a reduction of the quantity of GABA_A_ receptor can be found and a reduction of inhibition. In this case strengthening of conductivity of chlorine through GABA_A_ receptors can be considered as compensation for the reduced functioning of GABA systems.

### 3.3. Subunit Composition and Mutation of GABA_A_ Receptor

#### 3.3.1. Molecular Structure of GABA_A_ Receptor

GABA_A_ receptors concern an ionotropic super family of receptors representing a pentahedron [180]. Five subunits of GABA_A_ receptor surround the Cl^−^ channel [105–107, 181, 182]. GABA_A_ receptor subunits are a long polypeptide which penetrates a membrane in four places, that is why subunits possess four transmembrane domains. The so-called N- and C-terminals of the polypeptide are located on the extracellular surface. It is supposed that the second domain of GABA_A_ receptor subunit covers a wall of the chloride channel [183]. Between the third and fourth domains, there is a large intracellular fragment to which sites of protein kinase phosphorilation are concentrated and with which the basic differences in primary structure between various subunits are connected [181, 184]. On the N-terminal of the polypeptide leaving the first domain is the active center of a receptor or the sites connecting with ligands [105, 185–187].

Some specific binding sites are located on the GABA_A_ receptor [105, 182]. The first site is the GABA site where binding to GABA and its specific agonists such as gaboxadol, ibotenic acid, muscimol, and progabide takes place and to antagonists such as bicuculline and gabazine. Next there is the site of positive allosteric modulators such as barbiturates, benzodiazepines, carisoprodol, ethanol (alcohol), etomidate, glutethimide, kavalactones [188], meprobamate, methaqualone, neuroactive steroids [189, 190], niacin/niacinamide, nonbenzodiazepines, propofol, theamine, valerenic acid, and volatile/inhalation anesthetics. There is also the site of negative allosteric modulators such as flumazenil, Ro15-4513 and sarmazenil [191–193] and the site of noncompetitive channel blockers such as cicutoxin, oenanthotoxin, pentylenetetrazol, picrotoxin, and thujone. According to the definition, allosteric modulators which bind with GABA_A_ receptors in places which do not overlap with a binding site of an agonist, cause conformation reorganizations and change of receptor efficiency [194]. The chloride channel opens when two GABA molecules interact with their own site. Other sites only strengthen or reduce this interaction. GABA itself is an allosteric regulator for other sites. Next, the neurosteroid site will be discussed in more details. 

After interaction of neurosteroids with the GABA_A_ receptor, an intracellular process, oxygenation, is started; it transforms some intracellular metabolites in steroid receptors ligands [116]. After binding of these ligands with intracellular steroid receptors, the gene expression is modified. As the GABA site is an allosteric modulator of neurosteroid site, the interaction of GABA with its own site, allosterically modified by the neurosteroid site, initiates a cascade of events leading to the start of signal transduction. The transduction signal started through receptors processes of intracellular phosphorilation which terminate in updating the expression of genes. It means that the GABA_A_ receptor is an ionotropic receptor, but that it also initiates simultaneously signal transduction. Why is signal transduction so important? The modification of genes expression is a unique mechanism of an encoding process called memory consolidation, that is the transfer of information from short-term into long-term memory. Hence, signal transduction plays an important role at the long-term storage of information, in learning and memory, in training, in neuroplasticity in general, and in pathological states.

The GABA_A_ receptor has 22 subunits: *α*-6 subtypes, *β*-4 subtypes, *γ*-3 subtypes, *δ*-1 subtype, *ε*-3 subtype, *π*-1 subtype, *ρ*-3 subtype, and *θ*-1 subtype [181, 195, 196]. It is necessary to notice that some isoforms of GABA_A_ receptor subunits have various variants. So, for *γ*2 subunit, two variants were revealed [197, 198], differing in length of an intracellular fragment between 3 and 4 domains. Similar distinctions are known for *β*2, *β*4 subunits for which the presence of two variants has also been shown [199, 200]. Each of such sequences (*γ*2, *β*2 and *β*4) has in the designation an additional letter L or S (accordingly, long and short) [181]. Two splays variant have been found for the *β*3 subunit [201]. It is supposed, those three splays variants exist for the *α*5 subunit [202]. A product of alternative splicing is the *α*6 subunit, truncated with an N-terminal [203]. The 18 types of GABA_A_ receptor subunits are expressed in a mammalian brain: *α* subunits (1–6), *β* subunits (1–3), *γ* subunits (1–3), *ε* subunits (1–3) and on 1 subunits *δ*, *π* and *σ* [204].

Different confirmations of the GABA_A_ receptor are found throughout the brain, and the most common mammalian subunits composition is two *α*1 subunits, two *β*2 subunits, and one *γ*2 subunit assembling in a pentahedron-(*α*1)_2_(*β*2)_2_(*γ*2). Two molecules of GABA interact with two sites, each of which is located on two N-terminuses from the first domain of *α*1 and *β*2 subunits. The benzodiazepine site is located on two N-terminus from the first domain of *α*1 and *γ*2 subunits. Efficiency of allosteric regulation depends on the subunit composition of GABA_A_ receptors [205].  Table 1 describes the degree of allosteric regulation. For example, 80% of brain GABA_A_ receptors are highly sensitive for diazepam, 10% of receptors have a low sensitivity for diazepam. The latter is the case if the subunit compositions of GABA_A_ receptor contain *γ*1 or *γ*3 subunits. The 10% of receptors not sensitive for diazepam have subunit compositions of GABA_A_ receptor containing *α*4 or *α*6 subunits. From the 80% of receptors that are highly sensitive for diazepam, 10% is insensitive for zolpidem. This is the case if the subunit composition of  GABA_A_  receptor contains a *α*5 subunit. The other 90% of receptors are zolpidem sensitive. The composition *α*1*β*2*γ*2 possesses high affinity for zolpidem, and compositions *α*2*β*3*γ*2 or *α*3*β*3*γ*2 demonstrate low affinity.

Absence epilepsy can be accompanied by modifications of GABA_A_ and GABA_B_ receptors subunit composition. Neonatal treated rats with AY9944 show an increased occurrence of SWDs which correlated significantly with a decreased protein expression of the GABA_A_ *γ*2 subunit in RTN and VB nuclei of the thalamus, but not in somatosensory cortex. Conversely, *α*1 subunit expression decreased in somatosensory cortex in the AY9944 model, but not in the thalamus [206]. The decreased GABA_A_ receptor *γ*2 subunits in thalamus and increased in cortex are gender and age specific [207]. These results coincide with data that in human absence epilepsy is more widespread among women. The synaptic physiology and network property of mice lacking GABA_A_ receptor *α*3, a subunit that is uniquely expressed in the thalamus by inhibitory neurons of the RTN, have been investigated. Deletion of this subunit produced a powerful compensatory gain in inhibitory postsynaptic response in the RTN. Although other forms of inhibitory and excitatory synaptic transmission in the circuit were unchanged, evoked thalamic oscillations were strongly dampened in *α*3 knockout mice. Furthermore, pharmacologically induced TC absence seizures displayed a reduction in length and power in *α*3 knockout mice. These studies highlight the role of GABAergic inhibitory strength within the RTN in maintenance of thalamic oscillations and demonstrate that inhibitory intra-RTN synapses are a critical control point for regulating higher order corticothalamocortical network activity [208]. The combination of immunocytochemistry and high-resolution immunogold electron microscopy was used to study cellular and subcellular localization of GABA_A_ receptors *α*1, *α*3, and *β*2/*β*3 subunits in ventral posterior nucleus (VP) and RTN of WAG/Rij and control Wistar rats. In control rats, *α*1 subunits were prominent at inhibitory synapses in VP and much less prominent in RTN; in contrast, the *α*3 subunit was highly evident at inhibitory synapses in RTN. *β*2/*β*3 subunits were evenly distributed at inhibitory synapses in both VP and RTN. Immunogold particles representing all subunits were concentrated at postsynaptic densities with no extra synaptic localization. Calculated mean number of particles for *α*1 subunit per postsynaptic density in nonepileptic VP was 6.1 ± 3.7, for *α*3 subunit in RTN, it was 6.6 ± 3.4, and for *β*2/*β*3 subunits in VP and RTN, the numbers were 3.7 ± 1.3 and 3.5 ± 1.2, respectively. In WAG/Rij rats, there was a specific loss of *α*3 subunit immunoreactivity at inhibitory synapses in RTN, without reduction in *α*3 subunit mRNA or significant change in immunostaining for other markers of RTN cell identity such as GABA or parvalbumin. *α*3 immunostaining in cortex was unchanged [47]. These combined results obtained in mice and rats allow us to assume that different combinations of subunit compositions of GABA_A_ receptor in the RTN may contribute to or are the reason for decreased strength of GABAergic inhibition. The functional consequence is that when the RTN is stimulated by the cortex, the thalamic resonance might be larger and SWDs might last longer. Importantly, the changes in subunit composition were already present in presymptomatic rats, suggesting that the changes in the RTN are not caused by the seizures but that they are a property of WAG/Rij rats.

The possibility that absence seizures cause alterations in GABA_A_ receptor (*α*1, *α*4, *β*2, and *γ*2) subunit gene expression in thalamic relay nuclei was also explored, in this case in an acute absence seizure model, the GHB model [209]. A marked increase in *α*1 mRNA and a corresponding decrease in *α*4 mRNA in thalamic relay nuclei 2–4 h after the onset of GHB-induced absence seizures (when the seizures were terminating) were detected. These changes were selective to these *α* isoforms as neither *β*2 nor *γ*2 mRNA changed following seizures and occurred only in thalamic relay nuclei but not in hippocampus. The alterations in *α*1 and *α*4 mRNA persisted until about 12 h, and by 24 h after the seizure-onset the mRNA levels normalized. Blocking GHB-seizures produced no change in the levels of *α*1 and *α*4 mRNA in thalamic relay nuclei, suggesting that seizures themselves were responsible for mRNA alterations.

The expression of mRNA of GABA_B_ receptor subunit was studied in WAG/Rij [210] and GAERS [211]. By using Real-Time PCR, the researchers from the Avoli group have found that mRNA levels for most GABA_B1_ subunits are diminished in epileptic WAG/Rij neocortex as compared with age-matched nonepileptic controls, whereas GABA_B2_ mRNA is unchanged. Next, it was investigated the cellular distribution of GABA_B1_ and GABA_B2_ subunits by confocal microscopy and it was discovered that GABA_B1_ subunits fail to localize in the distal dendrites of WAG/Rij neocortical pyramidal cells [210]. The authors propose that these alterations may contribute to neocortical hyperexcitability and thus to SWD generation in absence epilepsy. 

GABA_B_ receptor expression in the somatosensory cortex, VB, and RTN in GAERS was also investigated [211]. In situ hybridization results showed a significant increase in mRNA for GABA_B1_ in the somatosensory cortex and a decrease in the ventrobasal thalamic nucleus but not in the RTN. By contrast, the immunocytochemical data revealed an increased expression of both GABA_B1_ and GABA_B2_ receptor subunits in all regions examined, somatosensory cortex, ventrobasal thalamic nucleus and RTN. The main finding was an upregulation of GABA_B_ receptor protein in the corticothalamic circuit in GAERS compared to controls. The results, as obtained in GAERS [211] and WAG/Rij [210] rats seem rather opposite to each other, the hyperexcitability in WAG/Rij rats strain and a wide spread upregulation of GABA_B_ receptors. We have proposed (see Sections 3 and 4), that both of these processes cause the same reaction, increase of SWD. Perhaps the two opposing processes provide the same response in different strain of animals.

Mutations in inhibitory GABA_A_ receptor subunit genes (GABRA1, GABRB3, GABRG2, and GABRD) of human have been associated with genetic epilepsy syndromes including childhood absence epilepsy, juvenile myoclonic epilepsy, pure febrile seizures, generalized epilepsy with febrile seizures plus, and Dravet syndrome (DS)/severe myoclonic epilepsy in infancy. These mutations are found in both translated and untranslated gene regions and have been shown to affect the GABA_A_ receptors by altering receptor function and/or by impairing receptor biogenesis by multiple mechanisms including reducing subunit mRNA transcription or stability, impairing subunit folding, stability, or oligomerization and by inhibiting receptor trafficking [48, 49, 212–222]. Besides, it is supposed that the chloride channel gene CLCN2 may be a susceptibility locus in a subset of cases of childhood absence epilepsy [223].

To understand the effect of these mutations, surface targeting of GABA_A_ receptors was analyzed by subunit-specific immunofluorescent labeling of living cells. An involvement of the *γ*2-Arg-43 domain in the control of receptor assembly was revealed. This may have some consequences to the effect of the heterozygous *γ*2(R43Q) mutation leading to childhood absence epilepsy and febrile seizure [224].

Stargazer (stg) mutant mice fail to express stargazin (transmembrane AMPA receptor regulatory protein *γ*2 (TARP*γ*2)) and consequently experience absence seizure-like TCSWDs that pervade the hippocampal formation via the dentate gyrus. As in other seizure models, the dentate granule cells of stg develop elaborate reentrant axon collaterals and transiently overexpress brain-derived neurotrophic factor. It was investigated whether GABAergic parameters were affected by the stg mutation in this brain region. GABA_A_ receptor *α*4 and *β*3 subunits were consistently upregulated, GABA_A_ receptor delta expression appeared to be variably reduced, whereas GABA_A_ receptor *α*1, *β*2, and *γ*2 subunits and the GABA_A_ receptor synaptic anchoring protein gephyrin were essentially unaffected. It was established that the *α*4 *βγ*2 subunit-containing, flunitrazepam-insensitive subtype of GABA_A_ receptors, not normally a significant GABA_A_ receptor in dentate gyrus neurons, was strongly upregulated in stg gyrus neurons, apparently arising at the expense of extra synaptic *α*4 *βδ*-containing receptors. This change was associated with a reduction in neurosteroid-sensitive GABA_A_ receptor-mediated tonic current. This switch in GABA_A_ receptor subtypes was not reciprocated in the tottering mouse model of absence epilepsy implicating a unique, intrinsic adaptation of GABAergic networks in stg. Contrary to previous reports that suggested that TARP*γ*2 is expressed in the dentate, it was found that TARP*γ*2 was neither detected in stg nor in control dentate gyrus. It was reported that TARP*γ*8 is the principal TARP isoform found in the dentate gyrus and that its expression is compromised by the stg mutation. These effects on GABAergic parameters and TARP*γ*8 expression are likely to arise as a consequence of failed expression of TARP*γ*2 elsewhere in the brain, resulting in hyperexcitable inputs to the dentate [225].

The expression of one GABA_A_ receptor gene, GABABR1, is distinguished by the expression of multiple splice variants that encode different isoforms of the receptor. Two novel GABABR1 variants, GABABR1h (R1h) and GABABR1i (R1i), which appear to arise from alternative splicing of the GABABR1 gene, were identified. The expression of R1h and R1i is differentially regulated in brain and peripheral tissues, but expression is not altered in the brain of GAERS. Both the R1h and R1i variants exhibit a novel 80-bp insert downstream of exon 4 that is flanked by consensus splice sites, and both encode C-terminal-truncated proteins. The new insight into the family of GABABR1 variants gained from this study identifies exon 4 as a preferred locus, or as a hot spot for regulated splicing in the GABABR1 gene. This finding correlates with the microexonic nature of exon 4 (21 bp). Bioinformatic analysis of micro-exon 4 and its flanking pre-mRNA sequences has revealed multiple, potentially competitive, exonic splicing enhancers that provide a mechanistic basis for the preponderance of alternative splicing events at this locus. Conservation of GABABR1 micro-exon 4 across species suggests a conserved functional role, facilitating either N-terminal protein production or post-transcriptional gene regulation through regulated splicing coupled to transcript decay [226].

Mutations in the genes encoding the subunits of the low-threshold T-type Ca^2+^ channel (*I*_Ca,T_) in patients [44–46, 227–231] and also in GAERS but not in WAG/Rij rats, are probably associated with a dysfunction of pacemaker *I*_h_ channels in cortex and thalamus. First, it has been shown that the *I*_f_ pacemaker channel in the atrial sinus node functions in pair with the  (*I*_Ca,T_)  channel [232, 233]. The *I*_f_ pacemaker channel in the sinus node opens at −80 mV, and the low-threshold (*I*_Ca,T_) channel is activated at −90 mV and fully inactivated at +40 mV. Blockage of the  (*I*_Ca,T_)  channels by low concentrations of NiCl_2_ diminishes the spontaneous activity of the *I*_f_ channel but does not affect the  (*I*_Ca,T_)  channel (the L-type Ca^2+^ channel). Participation of the low-threshold  (*I*_Ca,T_)  channel in the regulation of SWDs has been shown in both WAG/Rij rats [234] and GAERS rats [75]. There are two centers of bursting neurons which are activated by hyperpolarization: the RTN [19–22] as the source for spindle oscillations and the other is localized in the somatosensory cortex, the site of origin of SWDs [6, 9, 13, 14]. The somatosensory cortex is reciprocally connected with the VPM, VPL, and Posterior nucleus of the thalamus, both the ascending and descending pathways from VPM and VPL give collaterals to the RTN, which inhibits thalamic neurons. Interestingly, exactly the thalamus [67] and pyramidal neurons of cortical layers II, IV, and V of the somatosensory cortex [73, 74, 81] contain hyperpolarization-activated pacemaker *I*_h_ channels. The presence of hyperpolarization-activated pacemaker *I*_h_ channels in the RTN makes them an interesting object in the discussion and analysis of normal and pathological behavioral and neurophysiologic processes and mechanisms.

Thus, one of the reasons of the occurrence absence epilepsy is the mutation of several genes coding of GABA_A_ receptors which causes abnormal functioning of the GABAergic system. This abnormal functioning of the GABAergic system and mutations of  (*I*_Ca,T_)  channel, which functions in a tandem with *I*_h_ channel, can lead to the occurrence of spontaneous SWDs in an EEG.

## 4. Excitatory Glutamatergic System

It had been shown some time ago that the well-known noncompetitive NMDA antagonist MK-801 reduced the number and mean duration of SWD; the drug caused also large behavioural abnormalities such as head waving and agitation jeopardizing the interpretation whether the SWDs were primarily or secondarily (via behavior) affected by this NMDA antagonist [235]. Intracerebroventricular (i.c.v.) injection of NMDA induced a dose dependently increase in the number of SWDs. The competitive NMDA antagonist 2-amino-7-phosphonoheptanoic acid (APH) caused a dose-dependent reduction in the number and mean duration of SWDs. The effect of NMDA was blocked completely by APH [236]. Finally, the anticonvulsant remacemide, a noncompetitive, low-affinity NMDA receptor antagonist and its major metabolite FPL 12495 after oral administration suppressed the number of SWDs in WAG/Rij rats [237]. Interestingly, the mean duration of the few SWDs that persisted, was prolonged. These results obtained after i.c.v. injections demonstrated unambiguously that the NMDA receptor is involved in the control of SWDs. The involvement of other glutamate receptors such as AMPA and kainate receptors was also studied by i.c.v. injections of AMPA, GDEE (glutamate diethyl ester, a non-NMDA receptor antagonists), kainic acid, and kynurenic acid in WAG/Rij rats [238]. EEG registrations showed that AMPA dose dependently increased absences while GDEE caused a dose-dependent decrease. All effects of GDEE could be blocked by an inactive AMPA dosage. Kainic acid had no effects on nonconvulsive seizures. Kynurenic acid decreased dose dependently the incidence of SWDs; this effect of kynurenic acid could be blocked by a nonconvulsive dosage of kainic acid. Next the interaction between NMDA and non-NMDA receptors was studied [239]. Compounds acting on NMDA (NMDA, APH) and non-NMDA (AMPA, GDEE, kainic acid, kynurenic acid) receptors were coinjected i.c.v. It appeared that the epilepsy increase, induced by the non-NMDA receptor agonist AMPA, and in a less obvious way, by kainic acid, could be blocked by the NMDA receptor antagonist APH. The effects of NMDA were completely blocked by the non-NMDA receptor antagonists GDEE and kynurenic acid. Kain, the kainate agonist, had no effect on the number of SWDs, but the antagonist kynurenic acid decreased the discharges. CNQX, another non-NMDA antagonist, also reduces the number of SWDs [240]. 

The glutamate release inhibitor and anticonvulsant lamotrigine thought to inhibit the excitatory neurotransmitter release was indicated for nonconvulsive seizures [241]. Only in a high dose lamotrigine reduced SWD in WAG/Rij rats, but in that dose side-effect such as abnormal locomotion, were also seen [242]. Interestingly, a recent double blind EEG multicentered controlled study confirmed the poor absence suppressive effects of lamotrigine in childhood absence epilepsy [243]. 

In other series of experiments, two noncompetitive AMPA receptor antagonists, CFM-2 and THIQ-10c demonstrated to antagonize generalized tonic-clonic seizures in different animal models [100], were evaluated in WAG/Rij rats. Animals were focally microinjected into specific brain areas of the corticothalamic circuit and EEGs were recorded. The focal microinjection of the two AMPA antagonists into thalamic nuclei (ventralis posteromedialis, RTN, ventralis posterolateralis) and forelimb region was, generally, not able to modify the occurrence of SWDs. However, both compounds were able to reduce the number and duration of SWDs dose dependently when microinjected into the perioral region of the primary somatosensory cortex. These findings suggest that AMPA plays a role in absence epilepsy and that it depends on the involvement of specific neuronal areas. In the next series of experiments the authors demonstrated that noncompetitive AMPA receptor antagonists alone might not be useful in the treatment of absence epilepsy because of their low therapeutic index [244]. A similar low therapeutic index was earlier also found with the AMPA antagonist 7,8-methylenedioxy-1-(4-aminophenyl)-4-methyl-3-acetyl-4,5-dihydro-2,3-benzodiazepine (LY 300164) [135]. Nevertheless, the results from Citraro et al. showed that the center in the perioral region of the primary somatosensory cortex takes the lead or has a more considerable position in generation SWDs and that the local strengthening of GABA_A_ receptor activity in RTN strengthens SWDs [99].

In some studies, the effects of glutamatergic receptors ligands cannot be explained by a simple interaction at the receptor. To illustrate, the effects of ketamine, a noncompetitive antagonist at the NMDA receptor, were studied on the EEG and in the open field in the WAG/Rij rat strain [245]. Ketamine was systemically administered in a dose range from 3 to 30 mg/kg. Biphasic effects of ketamine were observed in the EEG. The first phase was a dose dependent suppression of SWDs, followed by a second phase characterized by the facilitation of SWDs. This increase was expressed first as an increased number of SWDs, and later on as a significant prolongation of individual discharges and decrease in frequency of SWDs. An obvious amplitude modulation of the discharges was also found. Ketamine also produced a dose-related initial behavioral excitation, a decrease of muscle tone in hind quarters, followed by front quarters and head, and an absence of locomotor activity. However, the time course of the behavioral changes was not in parallel with the complex EEG effects. It can be concluded that ketamine has more effects on the EEG than previously assumed which cannot be explained by a simple blockade of the NMDA receptor. It is proposed that the obtained specific dynamics of SWDs' frequency may be caused by changes in the activity of the corticothalamocortical pacemakers that are generating SWDs. The following study was conducted to investigate the effects of two noncompetitive AMPA receptor antagonists, GYKI 52466 and GYKI 53405 (the racemate of talampanel, the latter drug is a potent and selective AMPA-receptor antagonist) on the generation of SWD parallel with the vigilance and behavioral changes in WAG/Rij rats [246]. Intraperitoneal administration of GYKI 52466 (1-[4-aminophenyl]-4-methyl-7,8-methylenedioxy-5H-2,3-benzodiazepine), the prototypic compound of the 2,3-benzodiazepine family acting as an ionotropic glutamate receptor antagonist, and which unlike the conventional 4,5-benzodiazepines, does not act on GABA_A_ receptors, caused a fast dose-dependent increase in the number and total duration of SWD. These changes were accompanied by dose-dependent increase in duration of light slow wave sleep and passive awake, vigilance states associated with the presence of SWD. In addition a short, transient behavioral activation occurred that was followed by strong ataxia and immobility, decrease of active wakefulness and increase in deep slow wave sleep. GYKI 53405 (7-acetyl-5-(4-aminophenyl)-8-methyl-8,9-dihydro-7H-1,3-dioxolo[4,5-b][2,3]benzodiazepine), the racemate of talampanel, 16 mg/kg, i.p. failed to affect any measure of SWD and vigilance. When used as a pretreatment, GYKI 52466 (10 mg/kg) slightly attenuated the SWD-promoting effects of the 5-HT_1A_ receptor agonist 8-OH-DPAT, and it decreased the cumulative duration and mean duration of the paroxysms.

Interest of researchers has turned into metabotropic glutamate receptors (mGluR's) since their effects are more subtle and allow for a more refined intervention than would be the case with the ionotropic glutamate receptors. MGluRs are positioned at synapses of the corticothalamocortical network crucial for the occurrence of SWDs. mGluRs form a family of eight subtypes subdivided into three groups on the basis of amino acid sequence, pharmacologic profile, and G-protein coupling [247–249]. Group I comprises mGlu1 and mGlu5 receptors, which are coupled to Gq proteins. Their activation stimulates polyphosphoinositide hydrolysis with ensuing formation of inositol-1,4,5-trisphosphate and diacylglycerol. The receptors of this group also regulate the activity of different types of Ca^2+^ and K^+^ channels [250]. Group II includes mGlu2 and mGlu3 receptors and they are coupled to Gi/Go proteins. They negatively modulate the activity of adenyl cyclase and voltage-sensitive Ca^2+^ channels (VSCCs). Group III comprises mGlu4, mGlu6, mGlu7, and mGlu8 receptors, which are also coupled to Gi/Go proteins. The individual receptor subtypes have a modulatory role on excitatory and inhibitory synaptic transmission in the corticothalamocortical circuitry. Until now, receptors from all three groups have been studied in WAG/Rij rats. 

Group I mGluRs are preferentially localized in the peripheral portion of the postsynaptic density of both TCand corticothalamic cells, where they generate excitatory responses and regulate mechanisms of synaptic plasticity. Group II and III mGluRs are found preferentially (albeit not exclusively) in presynaptic terminals, where they negatively regulate neurotransmitter release [251].

*Group I*. mGlu1a receptor expression is reduced in the VB nucleus of the thalamus and pharmacologic potentiation of mGlu1 receptors with the selective PAM, SYN119 (corresponding to Ro0711401), reduces the incidence of absence seizures, whereas treatment with the mGlu1 receptor NAM (JNJ16259685) exacerbates SWDs [248, 249]. 

The expression of mGlu5 receptors was increased in the somatosensory cortex and decreased in the VB. Interestingly, these changes preceded the onset of the epileptic phenotype. SWDs in symptomatic WAG/Rij rats were not influenced by pharmacological blockade of mGlu5 receptors with MTEP, but were significantly decreased by mGlu5 receptor potentiation with the novel enhancer, VU0360172-6 without affecting motor behaviour. The effect of VU0360172-6 was prevented by cotreatment with MTEP [252]. It seems that PAMs affecting Group I mGluRs might be interesting new targets against absence epilepsy. 

The outcomes do contrast with data obtained in the lethargic (lh/hl) mice model, where mGlu1 receptor blockade with the orthosteric antagonists, AIDA or LY367385, reduces the incidence of SWDs [253]. Therefore, it appears that the role of mGlu1 receptors in the regulation of pathologic SWDs is species/model dependent and may also depend on the genetic determinants of absence epilepsy.

*Group II*. Symptomatic WAG/Rij rats showed an increased expression of mGlu2/3 receptors in the ventrolateral regions of the somatosensory cortex, ventrobasal thalamic nuclei, and hippocampus, but not in the RTN and in the corpus striatum, as assessed by immunohistochemistry and Western blotting [254]. In contrast, mGlu2/3 receptor signalling was reduced in slices prepared from the somatosensory cortex of these symptomatic rats, as assessed by the ability of the agonist, LY379268, to inhibit forskolin-stimulated cAMP formation. It therefore seems that mGlu2/3 receptors are involved in the generation of SWDs and that an upregulation of these receptors in the somatosensory cortex might be involved in the pathogenesis of absence epilepsy. 

*Group III*. Studies towards the role of receptors of this category have been limited because of lack of specific agonists. They are, however, interesting for absence epilepsy considering that activation of these receptors reduces GABAergic inhibitory responses within the thalamus, possibly via a presynaptic mechanism [255–257]. In addition, they have a profound effect in reducing the corticothalamic input onto thalamic relay cells [258]. Interestingly, and accordingly, an increased incidence of SWDs has been found in WAG/Rij rats when treated with the selective mGlu4 receptor enhancer, N-phenyl-7-(hydroxyimino)cyclopropa[b]chromen-1a-carboxamide (PHCCC) [259]. In humans, the mGlu4 receptor gene is localized at a susceptibility locus for juvenile myoclonic epilepsy [260, 261], which is characterized by absence seizures in addition to myoclonus and tonic-clonic seizures. Another group-III mGlu receptor, the mGlu7 receptor, is also associated with the development of absence seizures. Mutant mice in which mGlu7 receptors failed to interact with PICK1 (protein interacting with C kinase 1) develop absence seizures [262, 263], as do mice injected with a peptide that disrupts the interaction between mGlu7 receptors and PICK1 [262]. The precise mechanism whereby an interaction between mGlu7 receptors and PICK1 restrains SWDs in the corticothalamic network remains to be determined.

Thus, despite of the somewhat atypical features of the action of some substances on SWDs, it is possible to conclude that the role of the glutamatergic system in absence epilepsy corresponds to its role in idiopathic convulsive epilepsy. Seizures typical for both nonconvulsive and convulsive epilepsy could be due to the strengthened functioning of the glutamatergic system, which might be considered as hyperexcitability [157, 264]. The results suggest that the three types of ionotropic excitatory glutamate receptors enhance SWDs and this alleviating influence can be explained as follows. The spike in the SWD appears as a rebound spike, in fact it is the low threshold Ca^2+^ spike (Figure 1(b)). It appears after the hyperpolarization phase when the gating mechanism of an *I*_h_ channel is activated. Then the spike or the depolarization returns again to the hyperpolarization phase, and the process is repeated. The effects of all three types of excitatory glutamate receptors consist in the enhancement of the depolarization phase, that is, in increasing the amplitude of the spike. A larger spike increases the amplitude of the hyperpolarization, which in turn increases the subsequent spike [265]. This process appears as enhanced SWDs in the EEG. Besides, it is known from classical neurophysiology [266] that intensification of depolarization enlarges the frequency of action potentials, which are indeed crowned on the Ca^2+^ spike. 

## 5. Dopaminergic System of the Brain

### 5.1. Structure and Function of Mesencephalon DA Systems

**Figure 7:**
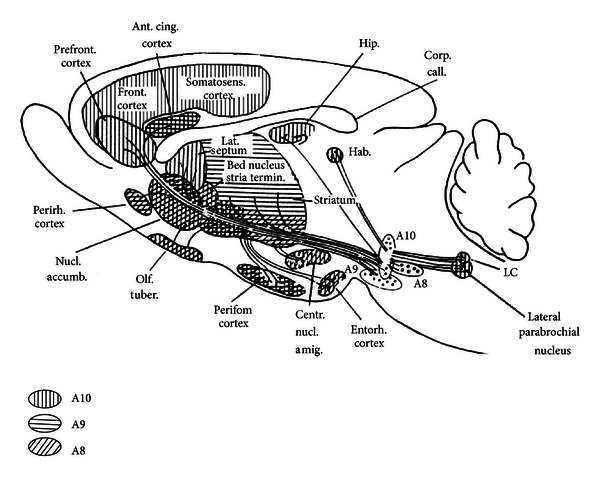
Schematic presentation of the efferent projections of the A8, A9, and A10 dopamine cell groups. The projection fields of the A10 neurons are designated by vertical hatching, those of the A9 cells by horizontal hatching, and the targets of the A8 DA neurons by diagonal hatching. Hatching is not intended to indicate the density of the various projections (scheme modified from [267]).

It is well known [267–269] that midbrain DA neurons are comprised of three groups of cells: A8, A9, and A10. The A10 DA neurons are located in the ventral tegmental area (VTA), whereas the more lateral DA cells of the substantia nigra pars compacta (SNpc) belong to the A9 cell group; the A8 neurons take a more caudal position in the retrorubral field (RRF) of the midbrain (Figure 7). The terminals of these neuron groups form the nigrostriatal and mesolimbic (or mesocorticolimbic) DAergic systems. A9 neurons group originating from SNpc predominantly forms the nigrostriatal system. The terminals of these cells innervate the caudate nucleus and neostriatum. The terminals of A10 VTA neurons innervate the mesolimbic system: hypothalamus, nucleus accumbens (Nab, the nucleus of the ventral striatum), olfactory tubercle, central and basal nucleus of amygdala, habenula, septum, hippocampus, prefrontal, cingulate, and entorhinal cortex. The mesocortical DAergic system is predominantly formed by A10 and partly by A8 neurons. The area of innervations by this system is the prefrontal, anterior cingulate, entorhinal, and piriform cortex and deep layer of the frontal cortex. The DA terminals innervate also the visual and motor cortex.

There are 5 types of metabotropic DA receptors associated with G proteins [270, 271] D_1_, D_2_, D_3_, D_4_, and D_5_. They belong to two basic families of receptors: the D_1_-like receptors including D_1_ and D_5_ DA receptors, the D_2_-like receptors including D_2_, D_3_, and D_4_ DA receptors. The D_1_-like receptors are associated with G_S_ proteins; they increase cyclic nucleotides (cAMP) synthesis and produce upregulation of DA-induced intracellular events such as phosphorilation leading to an alteration in cellular activity and changes in the gene expression within the responding cells. The D_2_-like receptors are associated with G_i_ proteins; they decrease cyclic nucleotides (cAMP) synthesis and produce downregulation of DA-induced intracellular reactions of dephosphorylation [272, 273]. Most of the D_2_-like presynaptic receptors are autoreceptors, an exception is the D_2_-like somatodendritic DA receptors [272–274]. These autoreceptors are located on DA terminals and downregulation occurs due to inhibition of DA synthesis and depolarization-induced release of DA from terminals [275–278].

**Figure 8:**
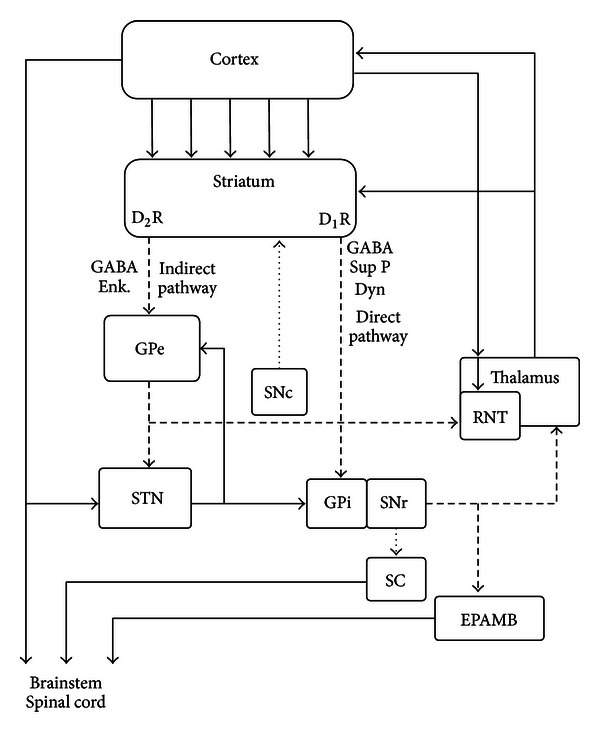
Communications of dorsal striatum. Continuous lines: glutamatergic communications (activating). Large dashed lines: GABAergic communications (inhibiting). Fine dotted line: dopaminergic communications. GPe: globus pallidus external. SNpc: substantia nigra pars compacta. STN: subthalamic nucleus. GPi: globus pallidus internal. SNpr: substantia nigra pars reticulata. RNT: reticular nucleus of thalamus. SC: superior colliculus. EPAMB: extrapyramidal area of a middle brain. Enk: enkephalin. Sub P: substance P: Dyn-dynorphin (scheme modified from [282]).

As specified above, the nigrostriatal DA system supervises the activity of GABA and glutamatergic synapses of the dorsal striatum (nucleus caudatus and putamen), which in turn activate TC networks and regulate motor behavior in animals and humans [279, 280]. Corticostriatal transmission is essential in the regulation of voluntary movement, in addition to behavioural control. Long-term potentiation (LTP) and long-term depression (LTD), the two main forms of synaptic plasticity, are both represented at corticostriatal synapses and strongly depend on the activation of DA receptors [273]. The dorsal striatum is extremely homogeneous in comparison with other cerebral areas. About 95% of dorsal striatal neurons have the same morphology and are medium spiny neurons, their function is to integrate all incoming signals [272, 279, 281]. The medium spiny dorsal striatal neurons receive excitatory glutamatergic synaptic information through AMPA, kainite, and NMDA receptors practically from all areas of the neocortex [272, 279]. The medium spiny neurons are GABAergic; they use GABA as synaptic transmitter. Consequently, postsynaptic inhibitory reactions are carried out through postsynaptic GABA_A_ receptors. Postsynaptic GABA_A_ receptors are located on dendrites and the soma of the striatal medium spiny neurons, and also on neurons of the globus pallidus, substantia nigra pars reticulate, and thalamus [279]. The medium spiny neurons of nucleus caudatus and putamen are morphologically identical, but differ neurochemically. One population contains GABA, dynorphin, and substance P and primarily expresses D_l_ receptors. These neurons send axons to GPi and to SNpr (Figure 8) and form a direct pathway which inhibit GPi and SNpr and are responsible for a reduction of an inhibitory influence on TC inputs, thereby activating TC input neurons. The second population contains GABA and enkephalin and primarily expresses D_2_ receptors. These neurons projects to GPe and form a indirect pathway which inhibits TC input (Figure 8). It is important to note that GABAergic terminals form an indirect pathway that project to neurons of the RTN [282]. That is, neurons of the RTN are controlled by D_2_ receptors through GABAergic projections from the ventral striatum.

In detail, an induction of intracellular signals DA by receptors and control of GABAergic and glutamatergic synaptic activity of medium spiny neurons of dorsal striatum has been described [272]. The DARPP-32/PP-1 (dopamine and cyclic adenosine 3′,5′-monophosphate-regulated phosphoprotein, 32 kDa/phosphoprotein-1) pathway integrates information from a variety of neurotransmitters and produces a coordinated response involving numerous downstream physiological effectors. DARPP-32 phosphorilation at Thr-34 by PKA is regulated by the actions of various neurotransmitters, principally DA acting at D_1_ receptors, but also adenosine acting at A_2A_ receptors and VIP acting at VIP receptors. PKG is activated in response to NO, also phosphorylates Thr-34 of DARPP-32. In medium spiny neurons that coexpress D_1_ and D_2_ classes of DA receptors, activation of D_2_ receptors acts to decrease cAMP levels. In medium spiny neurons that coexpress adenosine A_2A_ receptors and D_2_ receptors, activation of D_2_ receptors also acts to decrease cAMP levels. Opiates acting at either *μ* or *δ* receptors decrease cAMP levels in cells stimulated with D_1_ agonists or adenosine A_2A_ agonists, respectively. DARPP-32 phosphorylated at Thr-34 is dephosphorylated by PP-2B, a Ca^2+^/calmodulin-dependent protein phosphatase. PP-2B activity is activated by a number of different neurotransmitter receptors, principally following Ca^2+^ influx mediated by glutamate acting at NMDA receptors. Glutamate acting at AMPA receptors also stimulates DARPP-32 dephosphorilation by PP-2B, by an indirect mechanism that involves depolarization of the neuron and influx of Ca^2+^. Activation of D_2_ receptors also leads to an increase in Ca^2+^ levels, via an unidentified mechanism, and increased activity of PP-2B. In contrast, GABA acting at GABA_A_ receptors stimulates DARPP-32 phosphorilation by hyperpolarization of the neuron, decreased influx of Ca^2+^, and inactivation of PP-2B. Neurotensin acts to increase DARPP-32 phosphorilation by increasing the release of DA. CCK decreases DARPP-32 phosphorilation by increasing glutamatergic neurotransmission. The psychomotor stimulants cocaine and amphetamine increase DARPP-32 phosphorilation by increasing DA neurotransmission. All antipsychotic drugs achieve some of their clinical effects via antagonism of the D_2_ class of DA, an action leading to an increase in the state of phosphorilation of DARPP-32. Phospho-DARPP-32, by inhibiting the activity of PP-1, acts in a synergistic manner with different protein kinases to increase the level of phosphorilation of various downstream effector proteins. The resulting increase in phosphorilation is associated with increased activity of NMDA and AMPA glutamate receptors, L-, N-, and P-type Ca^2+^ channels, and early gene CREB and with decreased activity of GABA_A_ receptors, Na^+^ channels, and the Na^+^/K^+^-ATPase. It is important to note from [272] scheme that antagonists of the D_2_ class of DA receptors increase GABA_A_ receptors activity, and agonists of the D_2_ class of DA receptors decrease GABA_A_ receptors activity. Thus, intracellular integrating transduction signal modifies all parameters of neuronal activity. Modification of GABA and glutamate ionotropic receptors activity will lead to updating of synaptic neuronal inputs and outputs, defining participation of neuron in functioning of a neural network. Modification of voltage-dependent ionic channels activity modifies properties of the electrogenic neural membrane. Integrating transduction signal possesses the ability to modify not only the activity of ionotropic, but also of metabotropic receptors of neurons localized on membranes and supervising transduction signal. Such updating of all parameters of neurons, synaptic, electric, and chemical, switches neurons to another functionally active state or on other levels of functional activity. Depending on the brain structure and of the modulatory system inducing modification of transduction signal, a particular neuronal state can be formed which can switch into another functional state. For example, activation of the mesocorticolimbic DA system of ventral tegmental area (Figure 7, A10) lateral hypothalamus [283–285], nAcb [286, 287], prefrontal cortex [288–291], and anterior cingulate cortex [292, 293], induces an emotional positive state (pleasure and happiness [294–297]), a state referring to its own prolongation and/or intensification (intra-cranial-self-stimulation [283, 285, 286, 288, 290, 298], self-administration of drugs [290, 292, 299], self-control of drug administration, or place-preference paradigm [299–301]). Switching of neuron activity in other functional state, through intracellular integration with the help of transduction signal and modification of ionotropic and metabotropic receptors and potential sensitive ionic channels activity, it is possible to explain the role of the opioid [285, 298], GABAergic [284, 287], acetylcholinergic [289], serotoninergic [286, 287], and other systems in the control of the mesocorticolimbic DAergic reinforcement system. We define reinforcement as an emotional positive state arising as a consequence of a reward and of an avoidance or escape of an aversive stimulus, it induces purposeful motivated behavior.

As it has been shown above, one of the important structures of the mesocorticolimbic DA system is the nAcb, which is also called the ventral striatum [279]. The nAcb consists of two groups of cells which differ anatomically: the shell and core. Both parts of the ventral striatum participate in emotionally positive reactions and in reinforcement. The degree of participation of these two parts in reinforcement (see above) depends on features of the experimental model [302]. It is supposed that the core of the nAcb together with the dorsal striatum participates in the realization and control of purposeful behavior. The nigrostriatal DA system participates in regulation of motor behavior by regulation of TC neural networks (it is described in more details above). The shell receives innervations from A10 DA cellular groups (Figure 7), it is associated with the limbic system [268]. The nAcb core receives glutamatergic inputs from prefrontal cortices (another important structure of reinforcement reaction), thalamus, hippocampus, amygdala, and habenula. Direct and indirect projections from the core of the nAcb to the SNpr and VP in rats are described [303, 304]. Both structures receive projections from the dorsal striatum. Thus, the DA system of the ventral striatum (mesocorticolimbic) cooperates with that of the dorsal striatum (nigrostriatal) at the level of SNpr and VP which regulate TC networks through the GABAergic system. It is necessary to add that this interaction is supervised by the prefrontal cortex. It is the structural basis for the realization of purposeful motivated motor behavior, in particular when a choice for the correct response can be made in order to get reinforced. Behavioral experiments suggest that learning is driven by changes in the expectations about future salient events such as rewards and punishments. Physiological work has recently complemented these studies by identifying DA neurons in the primate whose fluctuating output signals changes or errors in the predictions of future salient and rewarding events [305–307].

### 5.2. Features of WAG/Rij Rats Strain Related to the Function of the DA System

It is known that SWDs are controlled by the brain's dopaminergic (DAergic) system. Antagonists of D_2_ DA receptors increase and agonists decrease of SWDs [32, 304, 308–310].

The WAG/Rij rats can be regarded as a valid genetic animal model of absence epilepsy with co morbidity of depression [50–52]. Behavioral studies indicate that WAG/Rij rats exhibit depression-like symptoms: decreased explorative activity in the open field test, increased immobility in the forced swimming test, and decreased sucrose consumption and preference (anhedonia). In addition, WAG/Rij rats adopt passive strategies in stressful situations, express some cognitive disturbances (reduced long-term memory), helplessness, and submissiveness, inability to make choices, and overcome obstacles, which are typical for depressed patients. Elevated anxiety is not a characteristic (specific) feature of WAG/Rij rats; it is a characteristic for only a substrain of WAG/Rij rats susceptible to audiogenic seizures [51]. Interestingly, WAG/Rij rats display a hyperresponse to amphetamine similar to anhedonic depressed patients. WAG/Rij rats are sensitive only to chronic, but not acute, antidepressant treatments, suggesting that WAG/Rij rats fulfill a criterion of predictive validity for a putative animal model of depression. However, more and different antidepressant drugs still await evaluation. Depression-like behavioral symptoms in this model are evident at baseline conditions, not exclusively after exposure to stress. Experiments with foot-shock stress do not point towards higher stress sensitivity at both behavioral and hormonal levels. However, freezing behavior (coping deficits) and blunted response of 5HT in the frontal cortex to uncontrollable sound stress, increased c-fos expression in the terminal regions of the mesocorticolimbic brain systems and larger DA response of the mesolimbic system to forced swim stress suggests that WAG/Rij rats are vulnerable to some, but not to all types of stressors. In all, it seems that the depressive-like behavior in WAG/Rij rats has a DA-dependent character [311]. These results allow the assumption that the WAG/Rij strain possesses a deficiency of the brain's DA system activity.

A low threshold for catalepsy, among others induced by sound stress, was repeatedly found in WAG/Rij compared to Wistar rats [308, 312]. WAG/Rij rats showed also both a larger novelty and amphetamine induced increase in total distance moved than ACI rats in a brightly lit open field [310]. This novelty/amphetamine-induced ambulation score was interpreted as an index of the reactivity of the DA system in the mesolimbic system [313–315]. Index of the reactivity is in addition bound, as well as a catalepsy threshold, to a threshold of sensitivity of a ligand-receptor interaction. It means that the dissociation constant (*K*_d_) of WAG/Rij rats DA receptors is lower than in nonepileptic rats [316]. Unfortunately, there are no data measuring the dissociation constant of DA receptors.

It is possible to assume that control of SWDs by D_2_-like DA receptors is carried out through an indirect GABAergic pathway from the dorsal striatum which terminates in the RTN through pathways passing through the GP (Figure 8), one of the sources of rhythmic oscillations. Antagonists of D_2_-like DA receptors enlarge activity of GABA_A_ receptor and increase SWD activity, and agonists reduce GABA_A_ receptor activity and reduce SWDs (see Section 3.1.1). D_2_-like DA receptors in dorsal striatum induces phosphorilation of GABA_A_ receptor and reduce its activity [272]. It has been shown that D_2_-like DA receptors selectively block N-type Ca^2+^ channels to reduce GABA release onto rat striatal cholinergic interneurons [317]. DA D_2_ receptor-mediated presynaptic inhibition of striatopallidal GABA_A_ IPSCs in vitro [318]. There were significant increases in [^3^H]nemonapride binding to D_2_-like receptors at different time points due to treatment with haloperidol, chlorpromazine, and olanzapine in rat striatum. By contrast, treatment with clozapine and olanzapine caused a time-dependent decrease in [^3^H]muscimol binding to the GABA_A_ receptor of striatum [319]. Dopamine D_4_ receptor induced postsynaptic inhibition of GABAergic currents in mouse GP neurons [320]. An increase of GABAergic neurotransmission in the striatum of mice lacking DA D_2_ receptors was found [321]. The inhibitory effects of the DA D_2_ receptor agonist quinpirole on GABA_A_-mediated spontaneous inhibitory postsynaptic currents correlate with the total number of D_2_ receptor sites in the striatum [322]. L-DOPA acting as D_2_-like agonist inhibits GABA release in the rat globus pallidus and induces turning behavior in rats with unilateral lesions of the DA innervation [323]. Quinelorane, a dopamine D_3_/D_2_ receptor agonist, reduces prepulse inhibition of startle and GABA efflux in the VP [324]. GABA release from pallidal terminals in the STN nucleus and in the RTN is inhibited by activation of D_4_ receptors [325]. Dopamine inhibits GABA transmission from the GP to the RTN via presynaptic D_4_ receptors [326, 327].

Thus, antagonists of D_2_-like DA receptors strengthen GABA transmission into striatum, GP and RTN and raise SWDs by promoting burst activity in the RTN, and agonists operate in an opposite direction. Based on the electrophysiological and lesion studies in WAG/Rij rats it is proposed that there is no direct source of SWD activity in the RTN, however, bursting neurons in the RTN are a necessary condition for maintenance and spreading of SWD [13, 20, 99].

#### 5.2.1. The Processes of Defensive Learning and Memory Controlled by DA System

An infringement of WAG/Rij rat's behavior was shown [51, 52, 328–331]. A decrease of memory reproduction, spontaneous catalepsy, low threshold of haloperidol-induced catalepsy, disturbances in maternal behavior, and symptoms of depression-like behavior were found in WAG/Rij rats. All these data can be explained by a DA deficit on the WAG/Rij rat's brain. The goal of the investigation [332, 333] was to study the possibility of WAG/Rij rat's behavior correction by pharmacological activation of DAergic system by disulfiram and L-3,4-dihydroxyphenylalanine (L-DOPA, the precursor of DA synthesis). Higher doses of disulfiram (100–300 mg/kg) block the efficiency of dopamine-*β*-hydroxyls, the enzyme involved in the synthesis of noradrenalin (NA) from DA. A lower dose of 25 mg/kg of disulfiram increases the DA content in the brain without change of NA and transforms animals with an emotional negative reaction to animals with an emotional positive reaction in the emotional resonance behavioral reaction test [334].

Rats were tested in a passive light avoidance and in a classical two-way active avoidance task and the effects of this dose of disulfiram on memory were investigated. The index of memory trace storage (*I*_MT_) was calculated as

(1) IMT=A2−A1A1×100%,

where *A*_1_ is the number of avoidance's in learning session on day 1, *A*_2_ is the number of avoidance's in the session on day 2 (both 50 trials).

There were three series of experiments. First series: disulfiram was administered to Wistar and WAG/Rij rats of the 1st group 4 hours before the 1st day learning session. Second series: L-DOPA (also 25 mg/kg i.p.) was administered to Wistar and WAG/Rij rats of the 2nd group 4 hours before the 1st learning session. In the 3rd series of experiment disulfiram was administered immediately after 1st learning session in Wistar and WAG/Rij rats. Control Wistar and WAG/Rij rats received the same volume of saline.

**Figure 9:**
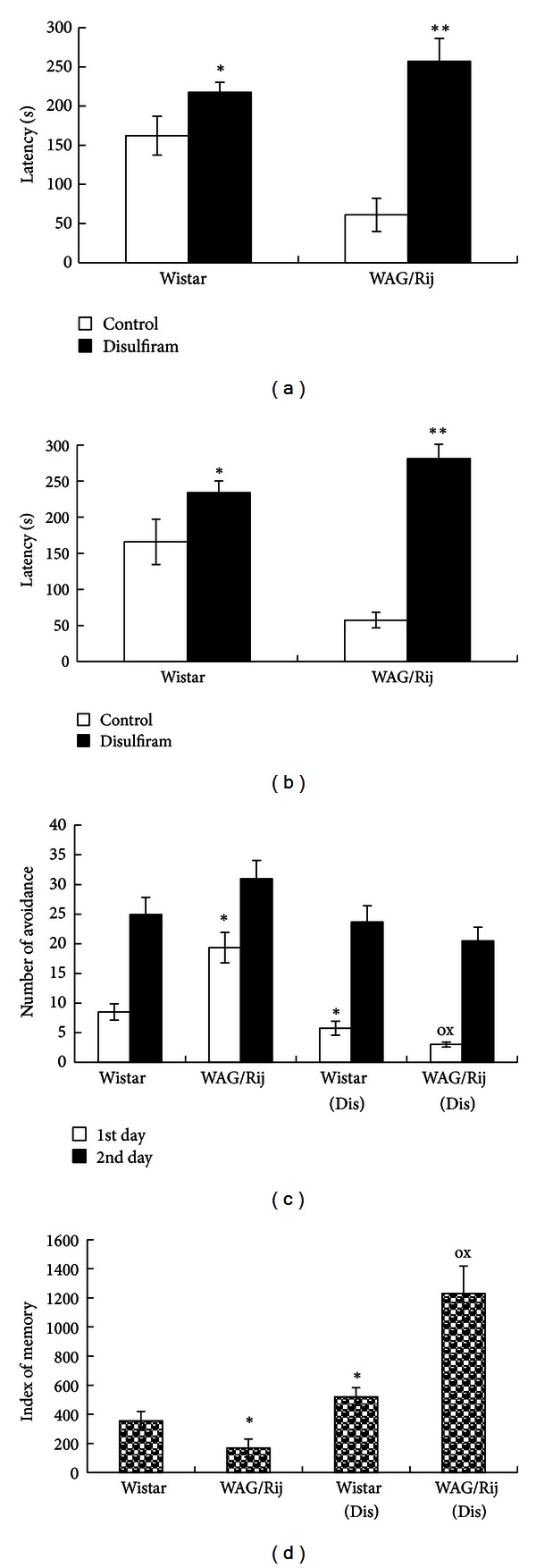
The action of disulfiram in the reproduction of passive and active avoidance reflex on the next day by Wistar and WAG/Rij rats strains. (a) is disulfiram (25 mg/kg) administration 4 hours before learning of passive avoidance. (b) is disulfiram (25 mg/kg) administration immediately after learning of passive avoidance. ^x^ *P* < 0.05 versus to Wistar, *P < 0.05 versus to control, **P < 0.01 versus to control. (c) is number of avoidances. (d) is index of memory trace storage. *P < 0.05 versus to Wistar control, ^x^ *P* < 0.05 versus to WAG/Rij control, ^o^ *P* < 0.05 versus to Wistar (Dis).

Control WAG/Rij rats showed an amnesic reaction compared to control Wistar rats at day 2 of the passive avoidance conditioning procedure (Figures 9(a) and 9(b); in 2.65 and 3.89 times in (a) and (b), resp.). The administration of disulfiram before as well as after passive avoidance conditioning improved memory as measured the next day both in Wistar and WAG/Rij rats, although the extent of increase was higher in WAG/Rij than in Wistar rats. The reproduction of memory, as established in the passive avoidance test, was increased 1.34 and 1.41 times in Wistar rats and 4.21 and 4.89 times in WAG/Rij rats accordingly. Administration of disulfiram before learning did not change the latency to enter the dark compartment in the first day nor induced a difference between the strains later.

The administration of disulfiram 4 hours before establishment of active avoidance conditioning changed the learning processes in the first day (Figure 9(c)). Control WAG/Rij rats realized 2.23 times more avoidance responses than control Wistar rats in the first day of learning (Figure 9(c)). Disulfiram administration (DIS) before learning decreased the number of avoidance responses in the first day: in Wistar rats 1.47 times and in WAG/Rij rats 6.45 times (Figure 9(c)). An amnesic effect in control WAG/Rij versus control Wistar rats was found in the second day of learning: the memory index of WAG/Rij rats was 2.11 times lower than in Wistar rats (Figure 9(d)). The administration of disulfiram increased memory index of Wistar rats 1.44 times and in WAG/Rij rats 7.33 times (Figure 9(d)).

**Figure 10:**
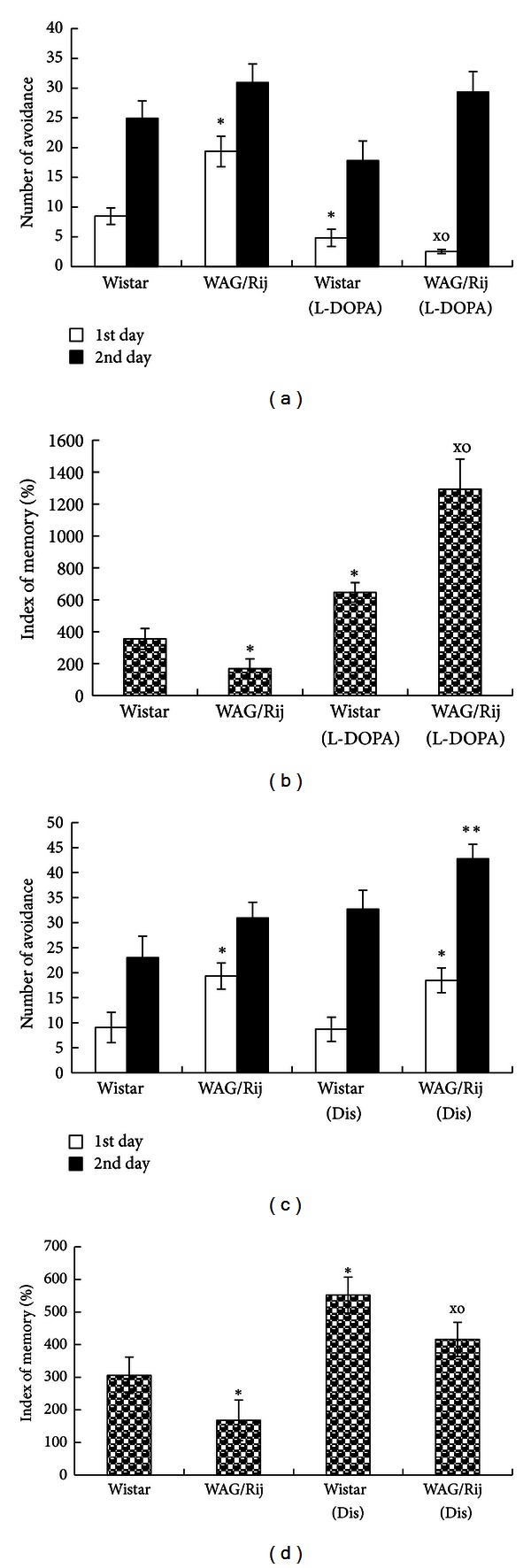
The action of administration of L-DOPA (25 mg/kg) and disulfiram (Dis) (25 mg/kg) on two days active avoidance conditioning sessions for Wistar and WAG/Rij rats. (a) and (b) are L-DOPA administration 4 hours before learning of active avoidance, *P < 0.05 versus Wistar control, ^x^ *P* < 0.05 versus WAG/Rij control, ^o^ *P* < 0.05 versus Wistar (L-DOPA). (c) and (d) of disulfiram administered immediately after the first day learning of active avoidance, *P < 0.05 versus Wistar, **P < 0.05 versus WAG/Rij and to Wistar DIS ^x^ *P* < 0.05 versus WAG/Rij control, ^o^ *P* < 0.05 versus Wistar (Dis).

The other inductor of DA system activation, L-DOPA, was used for comparison. Synergic effects of disulfiram and L-DOPA administered 4 hours before learning were found (Figure 10). The administration of L-DOPA 4 hours before learning decreased the number of avoidances in the first day of learning in Wistar rats 1.76 times and in WAG/Rij rats 7.65 times (Figure 10(a)). Herewith, the index of memory trace storage was increased in Wistar rats 1.82 times and in WAG/Rij rats 7.70 times (Figure 8(b)).

A higher number of active avoidance responses in the first day of learning and an amnesic effect in the second day of learning in WAG/Rij versus Wistar rats (Figures 9(c), 10(a), and 10(c)) was reproduced three times in our experiments. Administration of disulfiram immediately after active avoidance conditioning in the first day significantly increased the number of avoidance responses in the second day; the reaction of WAG/Rij rats was more intense than in Wistar rats (Figure 10(c)). In the memory trace storage the relation Wistar (Dis) to Wistar is 1.8 and relation WAG/Rij (Dis) to WAG/Rij is 2.48 (Figure 10(d)).

The amnesic effect in WAG/Rij versus Wistar rats in the passive avoidance reflex was found earlier [328, 335]. The amnesic effect can be explained by WAG/Rij rat's epileptic activity, the low efficiency of DA system and as a consequence, a reduced sensitivity for reward (see above). The improvement in the passive avoidance, (the dark was longer avoided) in both WAG/Rij and Wistar rats after disulfiram administration (Figure 7) can be explained by the same processes, which is an agreement with the changes in the emotional resonance behavioral reaction test [328]. It can be supposed that a low dose of disulfiram before as well as after learning increases the DA content in the brain, increase the sensitivity for reward, and, as a consequence, the consolidation process, the latter as expressed by a better performance in the passive avoidance test the next day. It is proposed here that the larger effects of disulfiram in WAG/Rij rats reflect their deficit in the DA system. Besides, the increase of DA system efficiency decreases in WAG/Rij rat's amount of epileptic activity [32, 304, 308, 309]. Activation of the DA system can be considered as an anti-amnesic effect. 

As it was specified above, the mesocorticolimbic DAergic system is a reinforcement system, not a reward system. We believe that there is a basic difference between reinforcement and reward. Usually, as reward food, sweets and so on are used. For some people one kind of food can be reward, and for other people the same food can be aversive. Hence, the relation to what is considered as reward is developed in the history of an individual's life. Usually reward, food and sweets are given through the mouth. As in the somatosensory and motor projections to dorsal striatum remains somatotopy inherent to the cortex [279, 280] it is natural that the reward involves nigrostriatal DA system [307, 336–340]. It is necessary to notice that a subset of striatal neurons (10%) is tonically active with discharge rates of 2 to 10 Hz. These neurons are distributed throughout the striatum and their discharge bears no specific relation to movement. These tonically active neurons (TANs) fire in relation to certain sensory stimuli that are associated with reward. For example, a TAN will fire in relation to a clicking sound if the click precedes a fruit juice reward but will not fire to the click alone or to the reward alone [279, 341]. It has been suggested that these neurons signal aspects of tasks that are related to learning and reinforcement. TANs are not activated by electrical stimulation of the GP and thus are probably not projection neurons. It is thought that TANs are cholinergic large aspiny interneurons that innervate the medium spiny neurons (see earlier discussions), which would place them in a position to modify the sensitivity of the medium spiny neurons to cortical input in relation to specific behavioral contexts [279].

We think that reinforcing properties of reward cause emotionally positive states which induce emotionally positive reactions, such as pleasure [342]. The purest kind of implication of the emotionally positive reaction refers to the prolongation of the emotionally positive state is the reaction of self-stimulation. Self-stimulation reaction can be observed only at stimulation of the mesocorticolimbic DA systems [291, 343–349], but not by stimulation of nigrostriatal DA systems. But prolongation of emotionally positive state is motivation, hence mesocorticolimbic DA system executes also function of motivation [348, 350]. Thus, the mesocorticolimbic DA system organizes behavior via modulation of emotional and motivation states. In this case, it is conceivable that tonically activated neurons of dorsal striatum play a complementary role, due to their small numbers. It is possible that these neurons are involved in the interaction processes of reinforcement and reward. We consider that in our investigation the avoidance of electrical current causes an emotionally positive state and reinforces an avoidance reaction. The augmentation of DA concentration in the brain by disulfiram or L-DOPA strengthens the reaction to a reinforcer. Against the prospect of a deficiency of DA and hyper reactivity of DA systems, the investigated substances provoke much more intensive effect in WAG/Rij than in Wistar rats.

The brain's opioid system also controls SWDs. Different agonists of kappa and antagonists of mu opioid receptors decrease SWDs, and mu receptors agonists increase SWDs [351–353]. It was also shown that a specific peptide of the proenkephalin system was increased in the striatum, the frontal cortex, and the mesencephalon [354, 355], structures directly or indirectly controlling inhibitory processes in corticothalamocortical neuronal networks [21, 279, 280]. Also from in situ hybridization studies, it appeared that the level of the proenkephalin mRNA was enhanced in the striatum of WAG/Rij rats [356].

The opioid system controls the threshold of pain sensitivity and actualizes the motivation of escape and avoidance of pain [299, 357, 358]. A high number of avoidance responses were found earlier in WAG/Rij rats on the first day of active avoidance conditioning (Figures 9 and 10) [328, 335]. We explain this reaction by the low efficiency of opioid system in WAG/Rij strain [351–356] and as a consequence a low pain threshold and a high level of escape and avoidance motivation. It has been shown [359–361] that activation of the DAergic system evokes analgesic reaction including activation of the opioid system [362–366]. It is important that in the analgesic reaction of DAergic system the nAcb is included [359, 360] and also that the interaction of DAergic and opioid systems takes place in the striatum [272, 364], a structure controlling SWDs. It is supposed that disulfiram or L-DOPA administration before learning increases the content of DA in the brain and via an enhancement of the opioid system activity increases the pain threshold and decreases pain avoidance motivation. At the same time DA-induced reinforcement (modification of mesocorticolimbic DA system) is increased, as well as memory consolidation and memory reproduction on the next day (Figures 9 and 10). It has been shown (Kuznetsova, not published data) that L-DOPA (25 mg/kg) administration decreases SWDs activity in WAG/Rij rats and that the same has been found for the DA agonists apomorphine and cocaine [32, 367].

Disulfiram administration immediately after learning increased DA-induced reinforcement (see above), increased the process of memory consolidation, and increased active avoidance memory reproduction on the next day (Figure 10). It is possible that the larger effect in WAG/Rij rats is a reflection of the DA and opioid systems efficiency deficit in this strain of rats.

In all, a deficit of DA system in WAG/Rij rats and an increase in the sensitivity to a DA agent as the response on the deficit is proposed to be the causative factor for the behavioural differences in Wistar and WAG/Rij rats.

A stable memory trace was found in the passive avoidance task in Wistar rats; however, WAG/Rijs performed less well. Disulfiram administration before and after learning increased memory reproduction in this task in both strains of rats. The enhancement of memory reproduction was more intense for WAG/Rij than for Wistar rats. WAG/Rij rats realized more correct avoidances than Wistar rats in the first day of active avoidance learning. But in the next day of learning a memory deficit was found in WAG/Rij rats. Low dose of disulfiram and L-DOPA administration before learning decreased learning in the first day, but increased active avoidance memory reproduction in the next day in both strains. This decrease of learning and enhancement of memory was more intense in WAG/Rij than in Wistar rats. It was suggested that a deficit of DA system in WAG/Rij rats is the biological correlate of these behavioural deficits and that an enhanced sensitivity to DAergic agents is the consequence of this deficit.

#### 5.2.2. Measurement of DA Systems Activity in WAG/Rij Rats Strain

Some parameters of DA activity in WAG/Rij rats were studied in order to establish whether there was a deficiency. DA synthesis and its degradation have been investigated in 70–80th of the last century in detail. It is well known that DA is synthesized in DA terminals from tyrosine. The limitation enzyme tyrosine-hydroxylase synthesizes L-DOPA (L-3,4-dihydroxyphenylanine) from tyrosine. The second enzyme, DOPA-decarboxylase, synthesizes DA from L-DOPA. DA is taken up by vesicles of varicosity and stored there [368, 369]. During depolarization, DA is released into the intercellular space and interacts with DA receptors. At this state a higher concentration of released DA, receptors activity and DA efficiency are observed. Further DA is retaken up in terminals and glia cells by assistance of DA-transporter. DA is inactivated by monoamine oxidase enzyme (MAO) and through a number of enzymatic transformations turns onto metabolite DOPAC (3,4-dihydroxyphenylacetic acid) measured experimentally. By the help of another enzyme of inactivationcatechol-O-methyltransferase (COMT) DA passes through another enzymatic transformations and turns into the metabolite HVA (homovanillic acid) also measured experimentally. The inhibition of the DA-transporter, MAO, and COMT activity reduces an inactivation of DA, reduces the concentration of DOPAC and HVA, raises the duration of DA interaction with receptors, and enhances the efficiency of the DA system [370–374].

The goal of our first experiment [375] was to investigate DA and its metabolites, DOPAC and HVA concentration in the following brain structures of Wistar and WAG/Rij rats: frontal cortex, parietal cortex, medulla, striatum, thalamus, and cerebellum. Concentrations of DA and its metabolites have been defined by method of High Performance Liquid Chromatography (HPLC). There was no difference in DA concentration in WAG/Rij compared to Wistar rats. However, the concentrations of metabolites and the HVA/DA ratio were substantially different in some structures between WAG/Rij and Wistar rats. DOPAC concentration was reduced in striatum and HVA concentration in thalamus in WAG/Rij rats. As noted above, one source of oscillations is located in thalamus [19–22]. The dorsal striatum is innervated by nigrostriatal DA system controlling activity of TC networks and modulates the occurrence of SWDs [21, 55, 279]. Reduction of the concentration of metabolites in thalamus and striatum is related to enhanced DA activity in these structures. The strengthening of DA activity may occur as compensation for DA deficiency at behavioural level.

The deficiency of DA activity is likely to be linked with changes of DA receptors. In order to test such probability, we compared [376, 377] D_1_ and D_2_/D_3_ DA receptors binding sites with autoradiography in brain areas of WAG/Rij while ACI rats were used as controls; the latter strain is nearly free of SWDs [79].

Analyses of the optical densities of the autoradiograms showed a significant reduction of [^3^H] SCH 23390 binding sites (D_1_ DA receptors) of WAG/Rij rats compared to ACI rats in the shell of nAcb and in the head of caudate nucleus. In other structures no significant changes were observed. The results are presented in Table 1.

The results of [^3^H] spiperone binding for D_2_/D_3_ DA receptors are given in Table 2. An increase of these binding sites was seen in WAG/Rij compared to ACI rats in motor, somatosensory, and parietal cortex. The number of binding sites was substantially lower than in the same structures of ACI rat; in the head of caudate nucleus and in the hippocampal CA3 area, a higher number was found in WAG/Rij rats. No significant differences in these measures were found in other structures.

Our results, a decrease of D_1_ DA receptors number in nAcb shell and in the head of caudate nucleus (Table 1), might be related to a reduction of an upregulating effect of DAergic transmission induced by phosphorilation. The increase in the number of D_2_/D_3_ DA receptors in motor, somatosensory, and parietal cortex was due to an amplification of downregulating effect of DAergic transmission, induced by dephosphorilation. The reduction of D_2_/D_3_ DA receptors number in the head caudate nucleus and in the CA3 area of hippocampus (Table 2) corresponds to reduction of downregulating effect of DAergic transmission, inducing the processes of dephosphorilation and enhancement of GABA_A_ receptors activity. The D_1_ DA receptors are somatodendritic receptors; they have not been found on DA terminals [378]. At the same time, D_2_/D_3_ DA receptors are both placed on soma and dendrites of target cells, and on DA terminals [272, 274–278]. As shown above, the autoreceptors modify DA release affecting a synthesis measured by DA concentration in tissue samples. In our previous study [375], we did not reveal significant differences in DA concentration in various brain structures of WAG/Rij compared to nonepileptic rats. It is possible that changes of D_2_/D_3_ DA receptors number revealed in this work were mainly related with somatodendritic receptors.

The low number of somatodendric D_2_-like DA receptors in the caudate nucleus induces endogenous processes of dephosphorilation and enhancement of GABA_A_ receptors activity in striatum, GP, and RTN (Figure 8) and is an endogenous cause for the increase of SWD activity in the corticothalamocortical network. The high number of somatodendric D_2_-like DA receptors in somatosensory cortex induce the endogenous processes of phosphorilation and decrease of GABA_A_ receptors activity in somatosensory cortex, forming an endogenous cause for decreasing SWDs in the cortical focus, which supervises an oscillatory drive in the RTN [20]. Possibly, it assumes compensatory reaction from outside somatosensory cortex in relation to caudate nucleus. That is why in WAG/Rij a compensated deficiency of D_2_-like DA receptors is observed. But D_2_-like DA receptors antagonists' administration both in cortex and caudate nucleus enhances processes of dephosphorilation and GABA_A_ receptors activity amplify, enlarging activity of SWDs.

A deficiency of mesolimbic (nAcb shell) DAergic activity at the level of somatodendritic D_1_-like DA receptors in WAG/Rij versus to ACI rats was found. At the same time a deficiency of nigrostriatal DAergic system in the head of caudate nucleus was caused by a reduction of D_1_-like and D_2_-like DA receptors number. The deficiency of nigrostriatal DAergic system by reduction of D_2_-like DA receptors is at the bottom of an additional rising activity of SWDs. As D_1_-like DA receptors supervise a direct pathway from striatum which terminates on non epileptic nucleus of thalamus (Figure 8), to us, there is no reason to assume that the deficiency D_1_-like DA receptors has an impact on the occurrence of activity of SWDs.

The deficiency of mesocorticolimbic DA systems corresponds to behavioral features of WAG/Rij rats. During active and passive avoidance a deficit of reinforcement in WAG/Rij rats has been revealed [332, 333] which was eliminated by administration of the DA precursor, or a low dose of disulfiram (Figures 9 and 10), inhibitor of dopamine-*β*-hydroxylase, by increase of DA concentration in brain [334]. Besides, it was shown that WAG/Rij rats have a higher level of depression than control Wistar rats without absence epilepsy [50–52, 379]. Depression of WAG/Rij rats has a DAergic nature [311] and can be explained by a deficiency of the DA mesolimbic system. Behavioural changes and activity of the mesolimbic DAergic systems in WAG/Rij rats are influenced by activation of D_1_ DA receptors in the shell of the nAcb [380, 381] and prefrontal cortex [382, 383], the main sources of positive reinforcement. D_2_-like DA receptors are also involved in reinforcement [380, 381, 384, 385]. A link between modification of behavior and alternative splicing of D_3_  DA receptor has been shown [386, 387]. The nAcb core [388] and neostriatum [381] are not involved in reinforcement. It is likely that D_1_- and D_2_-like DA receptors in the nAcb may have more general influence on reinforced behavior [381].

The nigrostriatal DA system mediates behavioral reactions through the control of TC neuronal networks. The nAcb shell receives DAergic cellular innervations from the VTA; this part of the nAcb is connected with limbic system [268, 279]. The nAcb core receives glutamatergic inputs from the prefrontal cortex and thalamus, as well as from hippocampus and amygdala. There are direct and indirect projections from nAcb core to SNpr and VP in rats [304]. The projections from ventral striatum also pass to these structures. Thus, interaction of mesocorticolimbic and striatonigral DA systems at level of SNpr and VP controls thalamocortical networks through GABAergic system, behavioral reactions, and absence epileptic activity [21, 304].

Despite a compensated deficit of DA system of WAG/Rij rats DA agonists and antagonists increase and decrease SWDs, respectively [32]. Similar results were obtained in the GAERS rats [389]. Intra-accumbal injections of DA agonists and antagonists produced increase and decrease of SWDs respectively [304]. Herewith the significant changes in the nAcb (upregulation of mRNA D_3_ DA receptor) were revealed, although the levels of mRNA coding transporters or enzyme of DA synthesis did not differ from controls [390]. The changes of DAergic system in nAcb core in GAERS rats were slightly different than those received in WAG/Rij rats at present work. But the general orientation of the regulation of SWDs by DAergic in GAERS and WAG/Rij rats was the same. Therefore it is possible to admit that the mechanisms of DAergic regulation are also similar. It can be that upregulation of mRNA D_3_ DA receptor in the nAcb also is a process of compensation.

Thus a deficiency in DA systems in WAG/Rij rats is described and is mediated by D_1_- and D_2_-like DA receptors. Reduction of D_1_-like DA receptors number in the shell of the nAcb and in nucleus caudatus, reduction of D_2_-like DA receptors number in nucleus caudatus and increase of D_2_-like DA receptors number in motor, somatosensory, and parietal cortex. Reduction of D_2_-like DA receptors number in nucleus caudatus enhances the activity of GABA_A_ receptors in striatum, GP, and RTN, causing a rise in SWD-activity in corticothalamocortical networks. High number of somatodendric D_2_-like DA receptors in somatosensory cortex inducing the endogenous processes of phosphorilation and decrease of GABA_A_ receptors activity in somatosensory cortex, causing endogenous decrease of SWDs in cortical focus, which supervises an oscillatory source in the RTN. We assume that it reflects compensated deficiency of D_2_-like DA receptors of WAG/Rij rats. Despite process of compensation antagonists of D_2_-like DA receptors can raise activity GABA_A_ receptors both in a somatosensory cortex and in the RTN, raising SWDs. Deficiency of WAG/Rij rats mesolimbic DA systems, caused by reduction D_1_-like DA receptors in the shell of the nAcb causes depression-like behavior of animals, weakens formation of emotional positive state, worsens processes of reinforcement, and counteracts learning and memory processes. Pharmacological intensification of DA systems takes out negative reaction of deficiency of mesolimbic DA system.

#### 5.2.3. Possible Mechanisms of Infringement of DA System Function in Absence Epilepsy

The question arises, if absence epilepsy is related to disturbances or mutations of GABA_A_ receptor and low-threshold T-type Ca^2+^ channel, then what is the role of DA receptors in it. Why is there a functional deficiency of these receptors? We suggest two mechanisms of infringement of the function of the DA system during absence epilepsy.

1. GABA_A_ receptors mediate trophic effectson embryonic brainstem monoamine neurons. GABA is present in the mammalian brain during early stages of development, where it may act as a trophic signal for developing neurons. In the embryonic rat, GABA axons project through the brainstem when 5-HT, DA, and NA neurons are being generated [391–394]. This raises the possibility that GABA exert trophic influences on developing monoamine neurons, if they express appropriate receptors. In adult rat brain, these neurons do express functional GABA_A_ receptors [395–397]. The initial disruption of GABA_A_ receptor disrupts trophic influences on developing monoamine neurons and induces pathological changes in monoaminergic systems in adult animals.
2. Intraneuronal integration triggered by a transduction signal. We believe that infringement of the function of the DA system by intraneuronal integration triggered by transduction signal occurs in the brain, especially in structures in which there are no DA neurons, but DA terminals, such as cortex, striatum, GP, thalamus, nucleus nAcb, and so on.

#### 5.2.4. Intraneuronal Integration Triggered by Transduction Signal

Earlier it has been shown [165] that the development of pentylenetetrazole (PTZ) kindling (4-month-old animals) is accompanied by 20% reduction of the density (*B*_max⁡_) of GABA_A_ receptor benzodiazepine (BDZ) sites. In 10-month animals, kindling reduced densities of the BDZ site of GABA_A_ receptor by 50%. Acute administration of a subconvulsive dose of PTZ in the rats at 10 months of age that were kindled at 4 month induces convulsive seizures and densities of GABA_A_ receptor BDZ site (*B*_max⁡_) to 20% of control rats. The results suggest that the density of the BDZ site at the GABA_A_ receptors is reproduced at a level of receptor molecule density independent of their current status and age. The increased density of BDZ site of GABA_A_ receptors in kindled versus control rats can be interpreted as an enhancement of GABAergic inhibition, while it is thought that seizures are based on the process of neuronal hyperactivation accompanied by a reduction in the BDZ site density both in kindling-induced and single-dose PTZ-induced seizures [165]. Therefore, it is likely that at 10 months, when seizures are retrieved by a PTZ challenge and the level of GABAergic inhibition is restored, the level of glutamate receptors may also be restored, assuming that they were modified and consolidated in the process of kindling 6 months ago. The level of glutamate receptors may only be restored if neuronal GABA and glutamate receptors interact within a single integrated system interconnected through an intracellular transduction signal.

The interaction and integration of neuronal GABA and glutamate receptors have been shown in several studies. A reduction in GABAergic functions in PTZ-induced kindling is blocked by MK-801, an antagonist of NMDA receptors [162]. NMDA receptors are also involved in the process of kindling induced by FG 7142, an inverse agonist of the BDZ receptor [398]. Kindling produced by high-frequency hippocampal stimulation induces an enhancement of NMDA receptors [399] as well as modification of GABA_A_ receptors [400]. Also, the NMDA-induced LTP is found to be controlled by the intercellular metabolic systems of the GABA_A_ receptor complex, being inhibited by agonists acting at the BDZ site [401, 402] and facilitated by its antagonists [403, 404]. Conversely, the NMDA antagonist CPP can modulate the long-term modification (tolerance) of BDZ receptors [405]. Positive allosteric activation of GABA_A_ receptors bi-directionally modulates hippocampal glutamate plasticity and behavior [406]. Finally, BDZ withdrawal anxiety is associated with potentiation of AMPA receptor currents in hippocampal CA1 pyramidal neurons attributable to increased synaptic incorporation of GluA1-containing AMPA receptors [407].

The BDZ site is one of the metabolic sites of the GABA_A_ supramolecular receptor complex [408–412] which is subject to long-term modifications [165, 400, 413]. The next of the GABA_A_ receptor metabolic site is the neuroactive steroids site [116, 414]. LTP and LTD, like any long-term processes, are maintained by modifications of gene expression [399, 400, 415–418]. BDZ site, acting via the second messengers system and protein kinase C, can induce slow metabolic reactions in the cell and modify gene expression in various structures of rat brain [105, 408, 410, 419]. GABA_A_ receptors are known to be regulated by phosphorilation mediated by protein kinase C and G [272, 273, 420–422]. Substrate for phosphorilation and dephosphorilation of GABA_A_ receptor is the intracellular loop between the 3rd and 4th domain [204, 423, 424]. 

**Figure 11:**
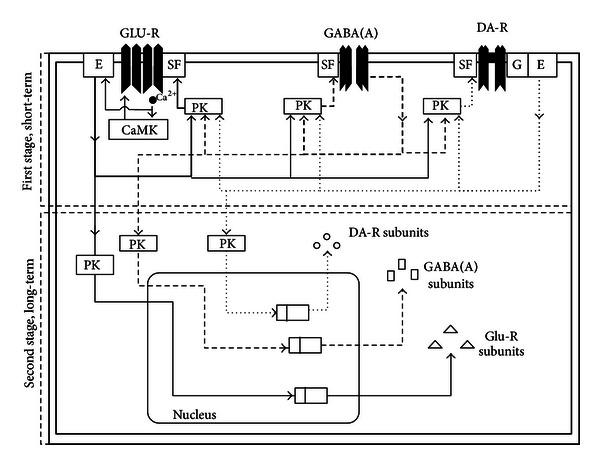
A tentative model of intraneuronal integration triggered by transduction signal. GLU-R: glutamatergic receptors; GABA(A), GABA_A_ receptor; DA-R: dopamine receptor; E: second messenger synthesizing enzyme; SF: phosphorilation substrate; G: G-protein, PK: different type of protein kinases; CaMK: calcium/calmodulin-dependent protein kinase. Metabolic reactions mediated by glutamatergic receptors are shown as *solid lines*. Metabolic reactions mediated by GABA_A_ receptor are shown as *dashed lines*. Metabolic reactions mediated by dopamine receptors are shown as *dotted lines*. Intracellular metabolic feedback loops (autoregulation) are shown as the respective *thick lines*. For other details, see text.

As indicated above, changes in cellular phosphorilation levels by protein kinases can modify glutamatergic receptors [162, 398, 399, 401–405], augmenting their responses to endogenous excitatory amino acids. The prospective scheme of this intracellular interaction is presented in Figure 11. The metabolic regulation of glutamatergic synapses (Figure 11) is described as a regulation of AMPA receptors by NMDA receptors. Control of AMPA receptor by NMDA receptor is described by plasticity in glutamatergic synapses of hippocampus, striatum, and cortex [273, 425]. We added a feedback loop for the regulation of AMPA receptors by NMDA receptors as it is repeatedly described in studies of the synthesis and trafficking of AMPA receptors subunit to the plasmatic membrane [426–428]. The regulation of AMPA receptors and autoregulation of NMDA receptors in the hippocampus, striatum and cortex have been studied experimentally [427, 429, 430]. The autoregulation of GABA_A_ receptors is also well described in investigations of synthesis and trafficking of GABA_A_ receptors subunit to the plasmatic membrane [204, 431–433].

Thus, one can assume that plasticity is a result of cooperative activity of GABA and glutamatergic receptors integrated into interrelated system. Integration includes also the automodification of the activity or receptors. Furthermore, a new level of activity, produced by secondary intranuclear signals, modifies the gene expression and consolidates the newly developed activity of receptors.

Functional interactions of the striatal glutamate and DA receptors in learning and memory [279, 305, 306, 434, 435], and postsynaptic integration of glutamatergic and DA signals in the striatum based on interactions of intracellular second messengers [272, 273, 279, 436–438] have been reported. The D_4_ receptor activation increases cofilin activity, causing a decrease in the myosin-based transport of GABA_A_ receptors clusters to the surface in the prefrontal cortex [439]. It has been shown [440] that the induction of LTD by low frequency stimulation in hippocampal CA_1_ neurons is under the influence of both NMDA and GABA receptors; both D_1_ and D_2_ receptors are involved in the modulation of LTD (activation of D_1_ receptors enhances LTD, activation of D_2_ receptors inhibits it) and the induction of LTD is blocked by picrotoxin, this effect is superseded by D_1_ receptors agonist SKF-38393. Our results [441] also point to synergistic interactions of GABA_A_ and DA receptors in memory retrieval.

The intracellular integration by transduction signal of glutamate, GABA_A_ and DA receptors is schematically shown in Figure 11. DA receptors can undergo automodification by the metabolic feedback loop and then modify the activity of glutamate and GABA_A_ receptors by intracellular phosphorilation [272, 273]. Via the same reactions of intracellular phosphorilation, glutamate and GABA_A_ receptors can control the efficiency of DA receptors. At the second stage, the modifications established at the first stage are consolidated through the modification of expression of the respective genes. The ability of DA receptors to undergo automodifications has been demonstrated both at the level of radioligand binding and at the level of gene expression in various brain structures and various experimental procedures [319, 442–447].

We suggest that diminished activity of DA receptors and DA system deficit occur due to disruption of intracellular integration triggered by a transduction signal (Figure 11). In the first place the initial disruption of GABA_A_ receptor activity disrupts the transduction signal from this receptor. Disruption of transduction signal shapes the modification of other receptors and their activity. In the second place the disrupted activity of receptors is consolidated and stored by expression of genes. It should be noted that we did not see changes of DA concentrations in the structures that we investigated, but we could see changes in receptor activity and consequences of receptors activity. So, a process of intracellular integration may disrupt activity of other neurotransmitter and neuromodulatory systems, for instance the activity of the opioid system that is disrupted in WAG/Rij rats [351–356].

If the first possible mechanism of infringement of the function of the DA system, mediation of trophic factor by GABA_A_ receptors, operates during a limited time period corresponding to the period of embryonic development, the second mechanism, intraneuronal integration triggered by transduction signal, operates during the whole life and, possibly, collects as a snow clot.

## 6. Conclusions

The hyperpolarization-activated and cAMP-modulated HCN channels contribute to direct rhythmic activation in the brain. Four isoforms of this channel are known: HCN1–HCN4. HCN channels resemble voltage-dependent K^+^ channels; they are tetrameric and consist of monomeric subunits, each containing six transmembrane domains (S1–S6) with a P-loop that forms a pore between the S5 and S6 domains. Different HCN subunits may be connected and form a heteromultimeric complex, thus significantly increasing the diversity of *I*_h_ channels. The HCN1 channel has a minimal response to cAMP binding (+4 mV) with a cAMP-binding domain, whereas the HCN2 channel has a clear response (+17 mV). The coexpression of HCN1 and HCN2 with heteromultimeric channel formation leads to *I*_h_ currents, which tend to possess functional properties intermediate between HCN1 and HCN2 homomeric channels. Experimental data suggest that the *I*_h_ channel and the low-threshold T-type Ca^2+^ channel (*I*_Ca,T_) work in tandem. Hyperpolarization opens the *I*_h_ channel and a cationic current depolarizes the membrane to the threshold and induces a spike. Hyperpolarization also opens the (*I*_Ca,T_) channel. The Ca^2+^ ions that enter the cell induce Ca^2+^-dependent cAMP synthesis and cAMP dramatically increases channel activity through binding to the CNBD locus of HCN subunits. The HCN1 subunit reacts weakly to cAMP binding; therefore, the decrease in the proportion of HCN1 subunits in the channel increases pacemaker activity and an increase in the proportion of HCN1 subunits in the channel decreases pacemaker activity. Analysis of the experimental data shows that one of the basic mechanisms for the long-term regulation of *I*_h_ pacemaker activity is the modification of the number of HCN1 subunits in the pacemaker channel. It has been assumed that the source of SWDs is the *I*_h_ pacemaker channel that is localized at pyramidal cells in the perioral region of the somatosensory cortex (in WAG/Rij rats), more specifically layers III-VI. This part of the cortex is fully integrated in a corticothalamocortical circuit, which intactness is obligatory for the full expression of the bilateral generalized SWDs. The RTN is part of this circuitry, it has also pacemaker properties, and it generates sleep spindles.

The density of a subtype of HCN channels (HCN1) in the apical dendrites of the subgranular layers in this onset zone determines the somatodendritic excitability and a decrease of these dendritic HCN1 channels neurons increases the occurrence of absences. Also in the thalamus HCN channels are involved in burst firing typical for delta activity. Burst firing of TC-cells is, however, rare during SWDs; it occurs in only 7% of the TC cells although it cannot be excluded that this a necessary condition for SWD occurrence. Burst firing is caused by tandem hyperpolarization of *I*_h_ and  (*I*_Ca,T_) channels.

Pharmacological observations supported this point of view since activation of GABAergic system through GABA_A_, GABA_B_ receptors and inhibition of GABA inactivation in the synaptic cleft all leads to unequivocal result, intensification of SWD activity. However, the measurements of the efficiency of GABAergic system lead to diverse results. There might be three reasons for this. First, different models of absence epilepsy such as GAERS and WAG/Rij rats, various types of mice with spontaneous mutations, and knock-out lines have been used. It is possible that in these different models, various mechanisms of GABAergic system deficiency are present. The second reason of occurrence of the variety of results is the so-called process of uncertainty of the central nervous system. The analysis of several works has shown that an increase or decrease of the general activity of the transmission system in different animals can be achieved in different ways and depends on specific features of these animals. The third reason of occurrence of diverse results can be processes of indemnification. Strengthening of conductivity of chlorine through GABA_A_ receptors as found in WAG/Rij rats can be considered as a compensation for the failure of inhibition of GABAergic systems.

One of the reasons for the occurrence of absence epilepsy is the mutation of several genes coding for GABA_A_ receptors which cause abnormal functioning of GABAergic system. Such abnormal functioning of the GABAergic system and mutations of  (*I*_Ca,T_) channel, which functions in tandem with *I*_h_ channel, can contribute to the occurrence of SWDs in the EEG.

However, SWDs during absence epilepsy may also be enhanced by the activation of the glutamate ionotropic and metabotropic excitatory system. Agonists of three type's ionotropic excitatory glutamate receptors AMPA, kainate and NMDA enhance SWDs; antagonists of these receptors reduce SWDs. It is known from classical neurophysiology that intensification of depolarization enlarges the frequency of action potential discharges. The metabotropic glutamatergic receptors also control SWDs, strengthening or controlling them, dependent on their location within the corticothalamocortical network. Various changes in number of receptors were identified within the network. Indeed some mGluR's enhance SWDs, while mGlu1 and mGlu5 receptor agonists and especially the positive allosteric modulators of Group I receptors might be good candidates to control absence seizures.

Deficiencies in DA systems in WAG/Rij rats were revealed. They are mediated by D_1_-  and D_2_-like DA receptors. Reduction of the number of D_1_-like DA receptors in the shell of the nucleus nAcb and in the nucleus caudatus, and a reduction of D_2_-like DA receptors number in nucleus caudatus and increase of D_2_-like DA receptors number in motor, somatosensory, and parietal cortex. Reduction of D_2_-like DA receptors number in nucleus caudatus causes an enhancement of GABA_A_ receptor activity in the striatum, GP, and RTN; they are responsible for sleep spindle generation and other rhythmic activity in TC networks. High number of somatodendric D_2_-like DA receptors in somatosensory cortex inducing the endogenous processes of phosphorilation and decrease of GABA_A_ receptors activity in somatosensory cortex, causing endogenous decreasing of SWD activity in the cortical focus, which might trigger and entrain thalamic rhythmic firing and oscillations in the form of SWDs. We assume that the enhanced D_2_-like DA receptors reflect a compensated deficiency. Despite process of compensation, antagonists of D_2_-like DA receptors can raise activity GABA_A_ receptors both in a somatosensory cortex and in the RTN, raising SWDs. Deficiency of WAG/Rij rats mesolimbic DA systems, caused by reduction D_1_-like DA receptors in the shell of the nucleus nAcb, causes depression-like behavior in animals, weakens the formation of emotional positive state, worsens processes of reinforcement and counteracts learning and memory processes. A pharmacological increase of the DA system removes the negative consequences of the deficiency of mesolimbic DA system.

The question arises, if absence epilepsy is due to disturbances or mutations of GABA_A_ receptors and low-threshold T-type Ca^2+^ channel what is the role of DA receptors in it. Why is there a functional deficiency of these receptors? We propose two mechanisms of infringement of the function of the DA system during absence epilepsy.

1. Intraneuronal integration is triggered by a transduction signal. We believe that the infringement of the function of the DA system by intraneuronal integration triggered by transduction signal is carried out in the brain, especially in structures in which there are no DA neurons, but DA terminals are present such as cortex, striatum, GP, thalamus, and nucleus nAcb.

We suggest that diminished activity of DA receptors and DA system deficits occur due to disruption of intracellular integration triggered by a transduction signal. The initial disruption of GABA_A_ receptor activity disrupts the transduction signal of this receptor in the first stage. Disruption of the transduction signal changes and modifies other receptors and their activity. On the second stage the disrupted activity of receptors is consolidated and stored by the expression of genes. It should be noted that we did not see changes of DA concentrations in structures investigated, but we could see the changes of receptors activity. So, a process of intracellular integration may disrupt activity of other neurotransmitter and neuromodulatory systems, for instance the activity of the opioid system is disrupted in WAG/Rij rats.

(2) GABA_A_ receptors mediate trophic effectson embryonic brainstem monoamine neurons. GABA is present in the mammalian brain during early stages of development, where it may act as a trophic signal for developing neurons. In the embryonic rat, GABA axons project through the brainstem when 5-HT, DA, and NA neurons are being generated. This raises the possibility that GABA could exert trophic influences on developing monoamine neurons, if they express the appropriate receptors. In adult rat brain, these neurons do express functional GABA_A_ receptors. The initial disruption of GABA_A_ receptor disrupts the trophic influences on developing monoamine neurons and induces pathological changes in monoaminergic systems of adult animals.

The first possible mechanism, intraneuronal integration triggered by transduction signal, operates during the whole life and, possibly, collects as a snow clod. The second mechanism, the infringement of the DA system's function on absence epilepsy and the mediation of trophic factor by GABA_A_ receptors, operates during a limited period of time corresponding to the period of embryonic development.

## Figures and Tables

**Table 1:** Density of [^3^H] SCH 23390 binding sites in different brain areas of ACI and WAG/Rij rats (D_1_ receptors).

Brain regions	ACI rats	WAG/Rij rats	*P* < 0.05
Nucleus accumbens (core)	7.12 ± 0.61	5.86 ± 0.79	
Nucleus accumbens (shell)	10.51 ± 0.33	8.96 ± 0.36	0.013
Caudate nucleus (head)	11.14 ± 0.25	8.35 ± 0.23	0.00004
Caudate nucleus (ventral)	11.09 ± 0.56	12.14 ± 0.67	
Caudate-putamen	7.75 ± 0.70	8.70 ± 0.36	

**Table 2:** Density of [^3^H] spiperone binding sites in different brain areas of ACI and WAG/Rij rats (D_2_/D_3_ receptors).

Brain regions	ACI rats	WAG/Rij rats	*P* < 0.05
Motor cortex	7.23 ± 0.52	9.8 ± 0.61	0.018
Somatosensory cortex	5.18 ± 0.32	8.19 ± 0.72	0.0052
Parietal cortex	6.41 ± 0.40	8.40 ± 0.78	0.0064
Nucleus accumbens (core)	16.15 ± 0.70	14.94 ± 1.25	
Nucleus accumbens (shell)	22.79 ± 0.54	21.79 ± 1.22	
Caudate nucleus (head)	25.89 ± 1.13	21.64 ± 0.47	0.0088
Caudate nucleus (ventral)	32.77 ± 1.09	36.33 ± 1.72	
Caudate-putamen	30.15 ± 2.77	27.87 ± 2.46	
CA3 area of hippocampus	2.80 ± 0.15	1.85 ± 0.30	0.024

## Abbreviations

ACI rats: Agouti Copenhague Irish rats
AMPA: 2-Amino-3-[3-hydroxy-5-methylisoxazol-4-yl] propionic acid
APH: 2-Amino-7-phosphonoheptanoic acid
BDZ: Benzodiazepine
cAMP: Cyclic adenosine mono phosphate
AY-9944: Cholesterol synthesis inhibitor Ayerst-9944
CCK: Cholecystokinin
CBZ: Carbamazepine
CD: Cyclodextrin
CFM-2: 1-[4-Aminophenyl]-3,5-dihydro-7,8-dimethoxy-4H-2,3-benzodiazepin-4-one
CNBD: Cyclic nucleotide-binding domain
CNQX: 6-Cyano-7-nitroquinoxaline-2,3-dione
COMP: Catechol-O-methyltransferase
CREB: cAMP-responsive element binding protein
CS^+^: Cesium
Cys: Cysteine
DARPP-32/PP-1: Dopamine and cyclic adenosine 3′,5′-monophosphate-regulated phosphoprotein, 32 kDa/phosphoprotein-1
DOPAC: 3,4-Dihydroxyphenylacetic acid
DS: Autosomal dominant
EEG: Electroencephalogram
GABA: Gamma-aminobutyric acid
GABRA1: The gene encoding the alpha 1 subunit of the GABA_A_ receptor
GABRB3: The gene encoding the beta 3 subunit of the GABA_A_ receptor
GABRG2: The gene encoding the gamma 2 subunit of the GABA_A_ receptor
GABRD: The gene encoding the sigma subunit of the GABA_A_ receptor
GAD: Glutamic acid decarboxylase
GAERS: Genetic absence epilepsy rats from Strasburg
GAT-1: Glutamic acid transporter-1
GAT-2: Glutamic acid transporter-2
GDEE: Glutamate diethyl ester
GHBA: Gamma-hydroxybutyric acid
GHB: Gamma-hydroxybutyrate
GHBL: Gamma-hydroxybutyrolactone
GIRK: G protein-gated inwardly rectifying K^+^
GPe: Globus pallidus external
GPi: Globus pallidus internal
GYG: Glycine-tyrosine-glycine
GYKI 52466: 1-[4-Aminophenyl]-4-methyl-7,8-methylenedioxy-5H-2,3-benzodiazepine
GYKI 53405: 7-Acetyl-5-[4-aminophenyl]-8-methyl-8,9-dihydro-7H-1,3-dioxolo [4,5-b][2,3]benzodiazepine
GVG: Gamma-vinyl GABA
HCN: Hyperpolarization-activated and cyclic nucleotide-gated
HPLC: High performance liquid chromatography
5-HT: 5-Hydroxytryptamine [serotonin]
HVA: Homovanillic acid
Ih/Ih: Mutant mice
IPSP: Inhibitory post synaptic potential
L-DOPA: L-dihydroxyphenylalanine
LGNd: Dorsal part of the lateral geniculate nucleus
LTP: Long-term potentiation
LTD: Long-term depression
MAO: Monoamine oxidase
mIPSC: Miniature inhibitory post synaptic current
nAcb: Nucleus accumbens
NA: Noradrenalin
NCSE: Nonconvulsive status epilepticus
NMDA: N-methyl-D-aspartate
NO: Nitric oxide
PCR: Polymerase chain reaction
PKA: Protein kinase A
PP-1: Phosphoprotein-1
PP-2B: Phosphoprotein-2B
PTx: Pertussis toxin
PTZ: Pentylenetetrazole
RRF: Retrorubral field
RTN: Reticular thalamic nucleus
SNpc: Substantia nigra pars compacta
stg: Stargazer
STN: Subthalamic nucleus
SWDs: Spike-wave discharges
TBPS: T-butylbicyclophosphorothionate
THIQ-10c: N-acetyl-1-[4-chlorophenyl]-6,7-dimethoxy-1,2,3,4-tetrahydroisoquinoline
THIP: 4,5,6,7-Tetrahydroisooxazolo(5,4-c) pyridin-3-ol
Thr-34: Threonine in the position 34
VB: Ventrobasal thalamus
VIP: Vasoactive intestinal peptide
VP: Ventral posterior nucleus
WAG/Rij: Wistar Albino Glaxo, originating from Rijswijk
ZD 7288: Non-selective *I*_h_ channel blocker.
